# Exploring *Trichoderma* diversity in the Western Ghats of India: phylogenetic analysis, metabolomics insights and biocontrol efficacy against Maydis Leaf Blight disease

**DOI:** 10.3389/fmicb.2024.1493272

**Published:** 2024-12-20

**Authors:** Nazia Manzar, Abhijeet Shankar Kashyap, Manish Roy, Pawan Kumar Sharma, Alok Kumar Srivastava

**Affiliations:** ^1^Plant Pathology Lab, ICAR-National Bureau of Agriculturally Important Microorganisms, Maunath Bhanjan, India; ^2^Molecular Biology Lab, ICAR-National Bureau of Agriculturally Important Microorganisms, Maunath Bhanjan, India

**Keywords:** galactopyranose, *Trichoderma* spp., secondary metabolites, host-pathogen interaction, diversity

## Abstract

The Western Ghats of India is recognized as one of the world’s eight “hottest hotspots” of biological diversity. *Trichoderma*—a well-known biocontrol agent, was explored from this hotspot. A total of 260 *Trichoderma* spp. isolates were studied, with 9% exhibiting strong biocontrol potential and crop growth-promoting activity. Furthermore, this study identified three novel isolates—*Trichoderma caribbaeum* var. *caribbaeum*, *Trichoderma lixii*, and *Trichoderma brevicompactum*—which are reported for the first time from the Western Ghats making a significant contribution to the field. Based on internal transcribed spacer ribosomal RNA (ITS-rRNA) and translation elongation factor 1-*α* (tef-1α) gene sequence analysis, molecular characterization, identified major isolates as *Trichoderma koningiopsis*, *Trichoderma asperellum*, *T*. *caribbaeum* var. *caribbaeum*, *T*. *lixii*, *T*. *brevicompactum*, *Trichoderma atroviride*, and *Trichoderma erinaceum*. Seed biopriming with the effective *Trichoderma* strain TR11 reduced the maydis leaf blight (MLB) disease index to 32.92% and improved plant growth-promoting attributes in maize. Defensive enzyme activities were increased 2.5–4.2-fold in various treatments with the TR11 isolate, along with enhanced lignification postpathogen inoculation, indicating strengthened plant defense mechanisms. The promising strain *T. brevicompactum*-TR11 produces secondary metabolites; among them, 5% were found to have a role in biocontrol activity such as octadecanoic acid, palmitic acid-TMS, 5-(4-nitrophenoxymethyl), furane-2-carboxaldehyde, and stearic acid-TMS, phosphoric acid-3TMS, galactopyranose, 5TMS. This study explored *Trichoderma* diversity in the Western Ghats of India with phylogenetic relationship, metabolomics insights, and biocontrol efficacy against MLB disease.

## Graphical abstract

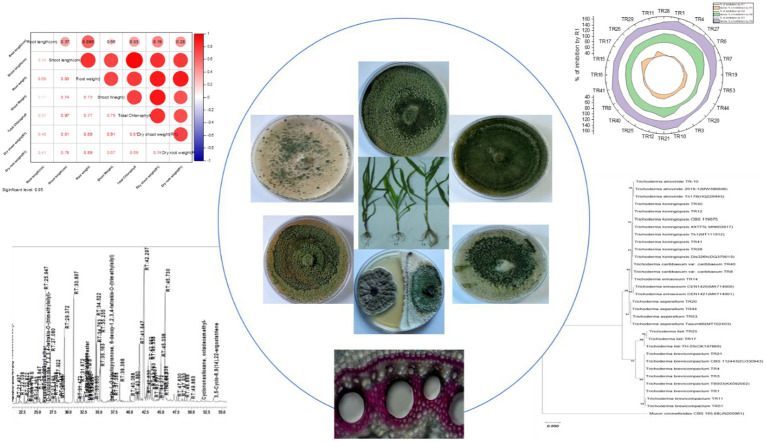

## Introduction

The Western Ghats of India—a UNESCO World Heritage site, is one of the world’s eight major biodiversity hotspots. It stretches from the southern tip of Gujarat through the Satpura range in the north, passing through Maharashtra, Goa, Karnataka, and Kerala, and ends at the southernmost part of India (Mehta and Shah, 2021). This region hosts a diverse range of organisms, including many novel and undiscovered microorganisms with significant potential for different applications (Nampoothiri et al., 2013). *Trichoderma*, common ascomycetes fungi found in soils globally, are widely studied for managing soil- and foliar-borne diseases. They are prevalent in various environments such as agricultural areas, grasslands, forests, and deserts (Jaklitsch, 2011). Multiple species of *Trichoderma* act as biocontrol agents against fungal pathogens (Li et al., 2013). A major threat is maydis leaf blight (MLB) or southern corn leaf blight (SCLB), caused by the fungus *Bipolaris maydis*, poses a significant threat to maize production globally (Manzar et al., 2022b). MLB can cause grain yield reductions of over 40%, with historical epidemics in the 1970s leading to losses of 28–91% (Zhu et al., 2021; Gogoi et al., 2014). Chemical control methods, though widely practiced, have several drawbacks, including environmental contamination, pathogen resistance, and adverse effects on non-target organisms. Hence, the use of biocontrol agents such as *Trichoderma* offers a sustainable and environmentally friendly alternative (Aggarwal et al., 2024). However, the efficacy of *Trichoderma* as a biocontrol agent can vary significantly among species and strains. Therefore, exploring the diversity of *Trichoderma* in different ecosystems and understanding their phylogenetic relationships is critical for identifying strains with superior biocontrol potential. Due to the lack of distinct morphological features and the rise of cryptic species, identifying *Trichoderma* based solely on morphology is challenging (Hoyos-Carvajal et al., 2009; Sun et al., 2012; Rodriguez et al., 2021; Manzar et al.,2022a). Molecular techniques now enable accurate identification of *Trichoderma* isolates and potential new species through gene sequence analysis (Druzhinina et al., 2005; Kubicek et al., 2008). The diverse genus Hypocrea *Trichoderma* includes around 160 species—the majority of them were identified via molecular phylogeny from pure cultures and herbaria specimens (Druzhinina et al., 2005). Surveys of *Trichoderma* spp. have been conducted in regions such as Siberia, the Himalayas (Kullnig et al., 2000), China (Zhang et al., 2005), Central and South America, and Egypt (Gherbawy et al., 2004). These studies identified unique species in each area and discovered new ones, possibly indicating species’ preference for specific regions or climates (Bissett et al., 2003; Kraus et al., 2004). The ability of *Trichoderma* spp. to induce resistance in maize plants against foliar pathogens has been proven (Harman, 2011; Jie et al., 2014). In response to pathogen attacks, plants exhibit various biochemical reactions, among which the antioxidative response holds significance due to the generation of numerous reactive oxygen species (ROS). This antioxidative defense mechanism involves the activation of enzymes such as peroxidase (POX), superoxide dismutase (SOD), and polyphenol oxidase (PPO). Plant pathogens trigger the induction of these enzymes through oxidative damage (Chugh et al., 2011). POX, a pivotal enzyme, participates in the biosynthesis of lignin and phytoalexins (Fan et al., 2006), and SOD operates to neutralize oxygen free radicals and active oxygen, thereby shielding plant membranes and essential enzymes within the salicylic acid pathway (Guo et al., 2006). *Trichoderma* spp. create a broad range of secondary metabolites that belong to many chemical groups, including alcohols, aldehydes, monoterpenes, sesquiterpenes, aromatic compounds, esters, furans, hydrocarbons, ketones, and compounds comprising S and N elements (Nieto-Jacobo et al., 2017; Esparza-Reynoso et al., 2021). These metabolites might involve in several biological activities, including microorganism-to-microorganism communication and biocontrol (Crutcher et al., 2013). The *Trichoderma* species are considered as plant growth-promoting fungi (PGPF), capable of colonizing and multiplying in the rhizosphere and strengthening plant defense systems. They can promote plant development in several indirect ways; however, little is known about metabolites-based interactions now. *Trichoderma* metabolites have been demonstrated to promote plant growth by acting as antibiotics against fungal pathogens (Vinale et al., 2014). This study aimed to ascertain the species diversity of *Trichoderma* gathered from the hot spot regions of Western Ghats of India and to find out the most potent *Trichoderma* isolates for managing MLB disease. This study focuses on characterizing *Trichoderma* isolates collected from the rhizosphere soil of various crops or plants in the Western Ghats region of India. The isolates were molecularly characterized and evaluated for their efficacy in reducing MLB severity caused by the pathogen *B. maydis*. Histochemical studies were conducted to assess disease reduction and to visualize lignin and callose deposition as the first line of defense, and also investigated the impact of these isolates on biochemical responses in maize plants, specifically the activity of defense enzymes (POX, SOD, and CAT), and analyzed secondary metabolites produced by the most effective *Trichoderma* isolates.

## Materials and methods

The study sampled various regions within the Western Ghats, including part of Shimoga, Coorg, Kodagu’s Somvarpet Taluk, Dakshina Kannada, Channarayapatna, Sakleshpur, Madikeri, Piriyapatna, Karwar, Begar, Sulya, and Udupi during an exploration survey conducted in December 2021. A total of 36 soil samples were collected, and the *Trichoderma* isolates were obtained and characterized in this study were obtained.

## Isolation and storage of pure cultures

Using the serial dilution plating approach, the *Trichoderma* selective medium described by Manzar and Singh (2020) was employed to isolate *Trichoderma* species. MgSO_4_·7H_2_O (0.2 g), K_2_HPO_4_ (0.9 g), KCl (015 g), NH_4_NO_3_ (1.0 g), glucose (3.0 g), Rose Bengal (0.15 g), and agar (20 g) make up 1 L of *Trichoderma* selective medium. Putative *Trichoderma* colonies were purified using potato dextrose agar (PDA) for two rounds of subculturing. Mineral oil was used to keep pure cultures at 4°C.

## Morphological analysis

Strains were cultivated on PDA for 7–8 days at 27°C to perform morphological examination using microscopic observations. After 3 days of incubation, pustules with white conidia were used to make microscopic slides for morphological features. To prepare the slides, a drop of 3% KOH was applied to each slide. KOH wets the conidia in the slide to provide better observation and aids in the conidiophores’ dissemination. According to Gams and Bissett, *Trichoderma* species were identified by Samuels (2006). Supplementary Table S1 lists microscopic observation and cultural morphology.

## DNA extraction and amplification

To extract DNA, Trichoderma isolates were grown on a PDA medium at 28°C for 4 days. After incubation, 3–4 mycelial plugs were transferred into a 250-ml conical flask containing 100 mL of potato dextrose (PD) broth, followed by shaking at 150 rpm at 28°C. The mycelial matrix was filtered using Whatman filter paper 42 following 6 days of development. It was cleaned using sterile distilled water and centrifuged at 10,000 rpm for 20 min at 4°C. Liquid nitrogen was then used in a pestle and mortar to pulverize it. The Nucleopore GDNA Fungus Kit-NP-7006D (Genetix Biotech Asia Pvt. Ltd., New Delhi, India) was utilized to extract genomic DNA (Manzar et al., 2021). Using a spectrophotometer (Shimadzu, Tokyo, Japan), the concentration and purity of the DNA were ascertained by measuring the absorbance at 260 and 280 nm. Using the primers ITS1 (TCCGTAGGTGAACCTGCGG) and ITS4 (TCCTCCGCTTATTGATATGC), the nuclear small-subunit rRNA gene’s ITS region was amplified in an automated thermocycler (Bio-rad-C1000 Thermal Cycler) by White et al.’s 1990 report. A 25-μl volume was used for the PCR, which contained 1X Taq Buffer with KCl (2.5 μL), 200 μM deoxynucleotide triphosphate (dNTP, 0.5 μL), 1.5-mM MgCl_2_ (1.5 μL), 1 U/reaction Taq polymerase (0.2 μL), 1X nuclease-free water (17.3 μL, Genetix brand), ITS 1 (10 pm) primer (1 μL), ITS4 (10 pm) primer (1 μL), and 100 ng genomic DNA (1 μL). The *Trichoderma* isolates ITS region was amplified at 95°C (initial denaturation) for 2 min, 40 denaturation cycles at 94°C for 1 min, 55°C (annealing) for 1 min, 72°C (extension) for 1 min, and at 72°C (final extension) for 10 min. Gel electrophoresis was performed for the polymerase chain reaction (PCR) products (10 μL), bands were recorded, and photographed in the gel documentation unit (Bio-Rad, Philadelphia, PA, USA). Bidirectionally Sanger sequencing method (Eurofins Pvt. Ltd) followed by sequence assembly with (Hall et al., 1999) was performed. For all ITS1, ITS4, and translation elongation factor 1-*α* (tef-1α) region sequences, multiple sequence alignment was performed using (Tamura et al., 2021) and CLUSTAL W (Thompson et al., 1994; Kashyap et al., 2023).

The ITS1 and ITS4 gene sequences of each *Trichoderma* isolate underwent alignment and basic local alignment search tool (BLAST) interface analysis using TrichoOKey, the National Center for Biotechnology Information (NCBI) database, and the TrichoBLAST program for tef-1α gene sequences. Sequence similarity percentages confirmed matches. Aligned sequences were then submitted to the DNA Data Bank of Japan (Mashima et al., 2017) and (Sayers et al., 2022) to obtain accession numbers for ITS and tef-1α regions. The molecular phylogenetic analysis employed the Maximum Likelihood technique using (Tamura et al., 2021) and (Tamura et al., 1993). A consensus tree was inferred, and nodal robustness was assessed using the bootstrap method with 1,000 replications to determine phylogenetic relationships among isolates and *Trichoderma* species.

### *In vitro* assessment of *Trichoderma* isolates for antagonistic activity against *Bipolaris maydis*

The test pathogen *B*. *maydis* and the 19 *Trichoderma* isolates were inoculated at the opposing corners of Petri plates to see if the isolates were antagonistic to each other (Morton and Stroube, 1955). *B*. *maydis* inoculation was performed 3 days ahead of the *Trichoderma* isolates since it developed slowly in a culture. The observations were reported on the sixth day after the dual culture experiment was run in three replications. The mycelial growth area of *B*. *maydis* was measured. Utilizing the formula described by Mokhtar and Aid (2013), the percent inhibition of the pathogen vis-à-vis the control was ascertained: mycelial growth inhibition (MGI) (%) = (C − T) × 100/C. Here, MGI represents the percent inhibition of mycelial growth, where C denotes pathogenic control, and T signifies the area of *B. maydis* mycelial growth in dual culture with *Trichoderma*.

### Inoculum preparation for maize plant treatment using selected bioagents

Water was used to soak the cleaned sorghum seeds for 12 h. The seeds were soaked, and then cooked for 20–25 min in a boiling water bath to soften the grains and then inoculated with a 3-day-old, actively growing *Trichoderma* isolate culture with the help of the sterilized aseptic cork borer, subsequently grown for 2 weeks at 28°C in a biochemical oxygen demand (BOD) incubator. The flasks were shaken rapidly to prevent clumping and disrupt the mycelial mat after 7 days of inoculation. The sorghum seeds that the *Trichoderma* isolates had colonized were allowed to air dry in the shade for 14 days before being pulverized in a mixer grinder.

Pure bioagent powder was obtained after being concurrently run through 50–80 mesh sieves. Talc powder and the finely ground *Trichoderma* isolates grown in sorghum seeds were combined in a 1:2 ratio, and the mixture was allowed to air dry for 3 days at room temperature in the shade. Approximately 5 g/kg of tapioca starch was added to the mixture. Pot tests were conducted using the final *Trichoderma* inoculum, which was adjusted in the formulation to 5 × 10^8^ cfu/g and then packed in polythene bags at room temperature (25 ± 2°C). In the fully grown culture of *B*. *maydis*, the conidia in the culture were scraped with the help of a spatula, and were suspended in deionized water containing 0.02% tween 20. This process produced the conidial suspension of *B*. *maydis*. As recommended by previous research, the final pathogen concentration was adjusted to 1 × 10^6^ conidia L^−1^ (Manzar et al., 2022b; Muimba-Kankolongo and Bergstrom, 2011). Using a hand-operated sprayer, the conidial suspension of *B*. *maydis* (10^6^ conidia L^−1^) was sprayed on plants that were 25-days old in the evening to induce leaf blight symptoms. Plants treated with water only were considered as control plants, and plants inoculated with *B. maydis* as pathogen-induced controls.

### Pot preparation and plant material

The glasshouse experimental setup comprised 5-kg plastic pots filled with sterilized autoclaved soil. Within each 30-cm plastic pot, healthy and surface-sterilized seeds of the susceptible Kanchan variety of maize were meticulously planted. The experiment included nine distinct treatments: T1 (CM212 + E7 + TR11), where CM212 is a moderately susceptible cultivar of maize, E7 is the virulent isolate of *B. maydis*, and TR11 is the isolate of *Trichoderma*; T7 (Dhiari local +TR11 + E7), where Dhiari local is the susceptible cultivar of maize, E7 is the virulent isolate of *B. maydis*, and TR11 is the isolate of *Trichoderma*; T4 (VL78 + E7 + TR11), where VL78 is the resistant cultivar of maize, E7 is the virulent isolate of *B. Maydis*, and TR11 is the isolate of *Trichoderma*; the pathogen inoculated control T8 (Dhiari local+E7), where Dhiari local is the susceptible cultivar of maize and E7 is the virulent isolate of *B. Maydis*; T2 (CM212 + E7), where CM212 is a moderately susceptible cultivar of maize and E7 is the virulent isolate of *B. Maydis*; T5 (VL78 + E7), where VL78 is the resistant cultivar of maize and E7 is the virulent isolate of *B. maydis*; the uninoculated untreated control T6 (VL78), where VL78 is the resistant cultivar of maize; T3 (CM212), where CM212 is a moderately susceptible cultivar of maize; and T9 (Dhiari local) where Dhiari local is the susceptible cultivar of maize. Ten seeds were sown per pot, with one healthy plant retained after germination. Each treatment was replicated five times in a completely randomized design. Pathogen inoculation occurred 25 days after sowing. Maize seeds underwent biopriming, involving surface sterilization with a 1% sodium hypochlorite solution followed by immersion in water for 24 h. Trichoderma-treated seeds received a talc-based formulation containing 5 × 10^8^ cfu/g @10 g/kg of seeds in a 2% gum arabic solution, following established protocols (Lewis et al., 1991). Samples were collected for each biochemical assay from individual treatments and from plants either inoculated with the pathogen or left uninoculated, at specific intervals postinoculation—namely, 0, 24, 48, and 72 h—to evaluate the induction of defense-related enzymes in maize plants under glasshouse conditions, with an average of three replications. The samples were stored at −80°C. Enzyme activity data, including assays for superoxide dismutase, POX, and polyphenol oxidase, were obtained using a spectrophotometer. SOD was estimated by the method described (Beauchamp and Fridovich, 1971). Polyphenol oxidase (PPO) activity was estimated by the method described (Mayer et al., 1966). POX activity was determined by the standard method (Hammerschmidt et al., 1982). Plant growth traits and disease severity were assessed 45 days after sowing. Disease severity was quantified using the percent disease index (PDI), calculated based on leaf area infection scores ranging from 1 to 9. The PDI was calculated using the following formula: PDI (%) = *Σ* (Scale × Amount of plants)/ (Maximum grade × Total number of plants) described by Wheeler (1969).

### Qualitative assay for ROS, callose deposition, and lignification

A maize leaves assay was conducted to measure reactive oxygen species (ROS) using the 3′,3′-diaminobenzidine (DAB) staining method. The DAB used in the assay was obtained from Sigma–Aldrich (USA), with catalog number D8001. The technique used in this assay was previously described by Thordal-Christensen et al. (1997), Bindschedler et al. (2006), and Daudi and O'Brien (2012). Leaf segments from various treatments were excised and submerged in a 1 mg/mL DAB solution (pH 3.8) for 8 h. This allowed the leaf segments to undergo a reaction with H_2_O_2_ and peroxidase. Leaf segments were treated with a solution containing 1.5 g/L trichloroacetic acid in ethanol:chloroform (4:1 v/v) for 48 h to achieve fixation. Subsequently, the segments were fixed in a solution consisting of 50% glycerol, which is commonly used in microscopy. Subsequently, digital photographs of leaf segments dyed with DAB were captured using a Canon digital camera from Japan. Each treatment consisted of four biological replicates. For callose deposition, leaves were cut into 5-mm strips; leaf samples were immersed in the fixative (ethanol/acetic acid, 3:1) and incubated for 12–24 h at room temperature. The fixed leaves were transferred into 1 N NaOH solution and incubate at 60°C for 1–2 h. Wash the cleared leaves several times with distilled water to remove NaOH. Stain the leaves by immersing them in aniline blue solution (in 1% [wt/vol]) aniline blue in 0.01 M K_3_PO_4_, pH 12, in darkness for 30–60 min in the dark at room temperature. After staining, wash the leaves gently with potassium phosphate buffer. The stained sections were mounted on slides and fixed using 95% ethanol before being observed under a compound light microscope manufactured by Nikon, Japan. The presence of blue color indicated callose deposition. The plants with different treatments were collected 15 days after being inoculated with *B. maydis* for lignin deposition. The roots were cut by hand and stained with a solution containing 1% phloroglucinol in 20% HCl. The stained sections were mounted on slides and fixed using 95% ethanol before being observed under a compound light microscope manufactured by Nikon, Japan. The presence of a pink color indicated lignin deposition, as described by Singh et al. (2011).

### Gas chromatography–mass spectrometry

Fungal culture filtrates were analyzed using gas chromatography–mass spectrometry (GC–MS) to detect active biomolecules. Secondary metabolites were identified using a single quadrupole mass spectrometer detector, following methods described by Stoppacher et al. (2010) and Siddiquee et al. (2012). The secondary metabolites in the culture filtrate of *Trichoderma* species were analyzed by GC–MS. Hydrocarbons and other secondary metabolites were separated from *Trichoderma* methanol extracts using a Gas Chromatograph GC-2020 (Shimadzu).

The mycelial mat from the petri plates was harvested. Liquid nitrogen grinding was carried out, and the obtained powder was collected into the tubes. A 6-ml metabolite extraction buffer (methanol:chloroform:water = 62.5:25:12.5) was added. Homogenization was carried out for 15–20 min. The sample was vortexed at retention time (RT) for 30 min. Centrifugation was performed at 13,000 rpm for 30 min at 4°C. After centrifugation, two layers were observed. The lower layer containing metabolites was taken and vacuum dried. To the pellet obtained, 100 μL of derivatizing reagent was added and incubated at 80°C for 30 min. *N*,*O*-Bis(trimethylsilyl) trifluoro acetamide + trimethylchlorosilane (BSTFA + TMCS) is used as a derivatization reagent for GC. This derivatized sample was further taken for GC–MS analysis; column: Spincotech DB-5 Column, flow rate: 1 mL/min, injection volume: 1 μL, run time: 60 min, and *m*/*z* range: 50–800 were used. The identified compound spectra were compared with those in the GC–MS database of the National Institute of Standards and Technology (NIST), with a threshold for detection set at over 90% resemblance.

### Statistical analysis

The data obtained from laboratory and glasshouse studies were analyzed using a one-way analysis of variance (ANOVA) method, as described by Trillas et al. (2006). Duncan’s multiple range test (DMRT) was employed to compare the means. The Statistical Product and Service Solution (SPSS) software version 16.0 was used for this analysis—created by SPSS, Inc., which is now known as IBM SPSS.

## Results

The exploration survey was conducted to study the *Trichoderma* spp. from the rhizospheric soil of the Western Ghats. A total of 260 *Trichoderma* isolates were obtained and subjected to morphological, biochemical, and molecular analyses.

### Morphological description

**Figure 1:**
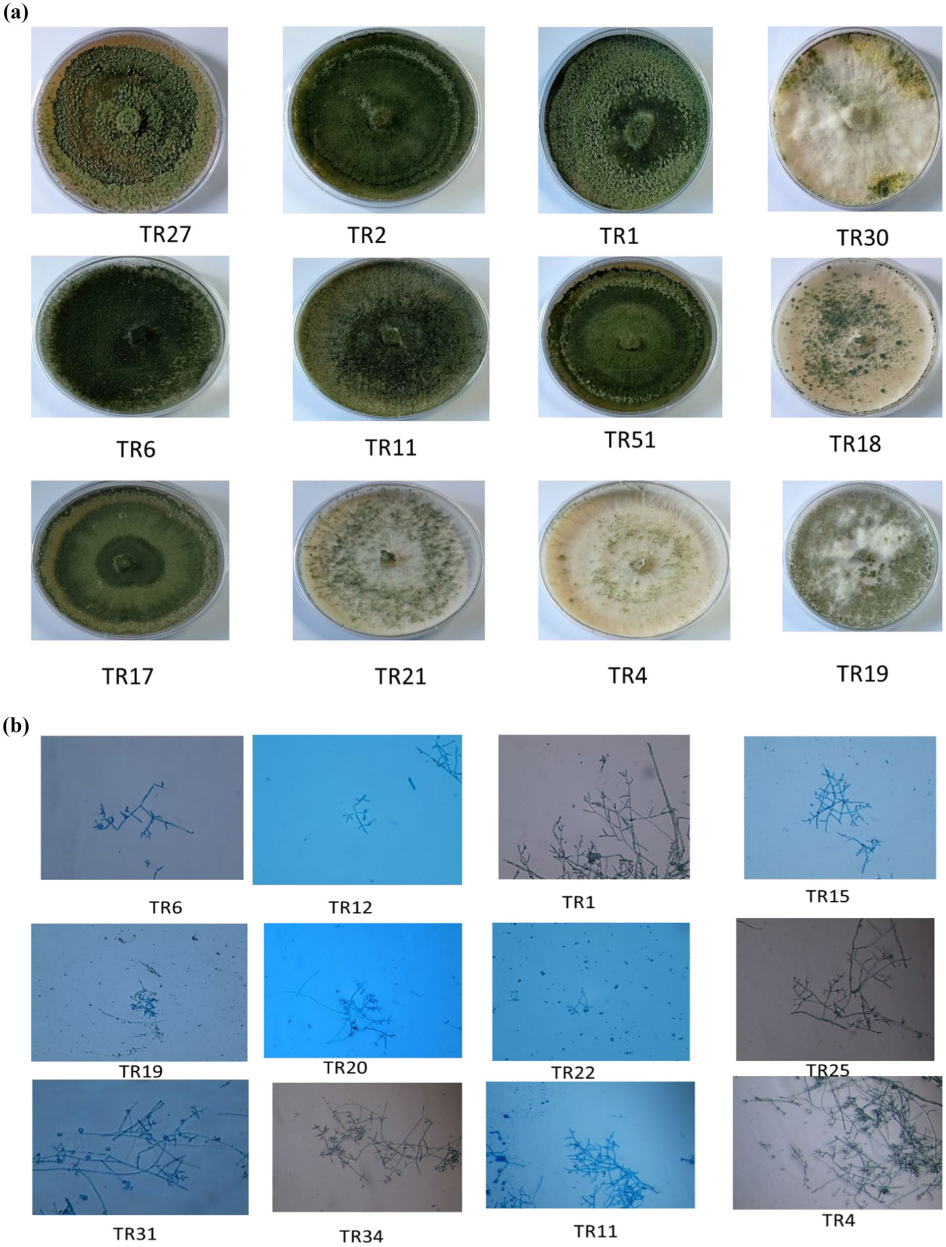
**(A)** Representative images showing the diverse colonies morphology of *Trichoderma* isolates on potato dextrose agar (PDA) media after 8 days of incubation at 27°C. **(B)** Microscopic characteristics of representative *Trichoderma* strains, showcasing phialides in groups emerging from small terminal clusters, conidiophores, and conidia (10× magnification).

Upon obtaining a pure isolate of *Trichoderma* spp., morphological characterization was conducted according to Barnett and Hunter’s (1972) method. This entailed assessing concentric rings, pigmentation, and mycelium texture, as detailed in Table 1 and illustrated in Figures 1A,B. Microscopic identification involved examining cultural structures such as phialides, hyphae, conidial shape, and conidiophore number. Conidia exhibited a mean length ranging from 3.4–3.5 μm and a mean width ranging from 2.7–3.0 μm.

**Table 1:** Detailing *Trichoderma* isolate data, including isolate codes, geographical information, host sources, genetic sequence accessions, and morphological characteristics of conidia.

Isolate code	GPS location	Country	Host	Source of isolation	GenBank accession no.		Identity of isolates	Conidia size Length × width	Conidia shape
					ITS	TEF			
TR41	13.87428 N 75.23412 E	India	Coconut	Rhizospheric soil	PP175190	LC799601	*Trichoderma koningiopsis*	3.7 μm × 2.3 μm	oblong to ellipsoidal or ovoidal and smooth
TR8	12.44 N 75.93 E	India	Bamboo	Rhizospheric soil	PP153914	LC799821	*Trichoderma caribbaeum var. caribbaeum*	4.8 μm × 2.5 μm	Conidia ellipsoidal to nearlyoblong, smooth.
TR40	12.43 N 75.89 E	India	*Artocarpus hirsutus*	Rhizospheric soil	PP153421	LC799822	*T. caribbaeum var. caribbaeum*	3.1–3.2 μm × 3 μm	Conidia ellipsoidal to nearly oblong, smooth
TR28	14.00 N 75.55 E	India	Arecanut	Rhizospheric soil	PP152293	LC799823	*T. koningiopsis*	3.9 μm × 2.7 μm	oblong to ellipsoidal or ovoidal and smooth
TR25	12.51 N 75.49 E	India	Rubber	Rhizospheric soil	PP141160	LC799824	*Trichoderma lixii*	3.5 μm × 1.9 μm	globose or subglobose conidia
TR12	12.92 N 76.40 E	India	*Curcuma longa*	Rhizospheric soil	PP141036	LC799825	*T. koningiopsis*	2.7 μm × 2.1 μm	oblong to ellipsoidal or ovoidal and smooth
TR21	12.93 N 75.76 E	India	Sugarcane	Rhizospheric soil	PP140921	LC800024	*Trichoderma brevicompactum*	2.7–3.5 μm × 2.5–3.0 μm	Conidia were sub-spherical or oval and smooth
TR-10	12.47 N 75.59 E	India	*Piper nigrum*	Rhizospheric soil	PP140887	LC800025	*Trichoderma atroviride*	3.0–4.0 μm × 2.0–3.0 μm	Conidia were sub-globose to globose, smooth, and thick-walled
TR30	12.41 N 75.71 E	India	Coffee	Rhizospheric soil	PP140873	LC800026	*T. koningiopsis*	3.9 μm × 2.5 μm	oblong to ellipsoidal or ovoidal and smooth
TR3	12.39 N 75.87 E	India	Senegalia catechu	Rhizospheric soil	PP140845	LC800027	*T. brevicompactum*	3.2 μm × 2.8 μm	Conidia were sub-spherical or oval and smooth, conidiophores was 1.8–2.9 × 0.9–2.1 μm
TR1	12.51 N 75.50 E	India	Rubber	Rhizospheric soil	PP140831	LC800028	*T. brevicompactum*	3.1 μm × 2.9 μm	Conidia were sub-spherical or oval and smooth
TR4	12.33443 N 76.1507 E	India	Tobacco	Rhizospheric soil	PP112347	LC800245	*T. brevicompactum*	3.5 μm × 3.0 μm	Conidia were sub-spherical or oval and smooth
TR17	12.33 N 76.15 E	India	Ragi	Rhizospheric soil	PP112276	LC800246	*T. lixii*	3.5 *μm* ×2.1 *μm*	globose or subglobose conidia
Tr14	12.42 N 77.20 E	India	CocoaCoconut	Rhizospheric soil	PP112272	LC800247	*Trichoderma erinaceum*	1.3 μm × 1.5 μm	ellipsoidal to broadly ellipsoidal
TR20	14.91 N 74.25 E	India	Maize	Rhizospheric soil	PP217768	LC800357	*Trichoderma asperellum*	3.32 μm × 3.18 μm	*globose toovoidal*
TR44	13.50 N 75.21 E	India	Rubber	Rhizospheric soil	PP229207	LC800358	*T. asperellum*	2.6 μm × 2.32 μm	*globose toovoidal*
TR53	15.02 N 74.77 E	India	Ragi	Rhizospheric soil	PP229206	LC800359	*T. asperellum*	3.09 μm × 2.95 μm	*globose toovoidal*
TR11	12.512045 N 75.509370 E	India	Rubber	Rhizospheric soil	PP346030	LC800360	*T. brevicompactum*	3.5 μm × 3.0 μm	Conidia were sub-spherical or oval and smooth
TR51	13.34 N 75.00E	India	Rubber	Rhizospheric soil	PP340179	LC800361	*T. brevicompactum*	2.8 μm × 2.1 μm	Conidia were sub-spherical or oval and smooth

### Molecular characterization of *Trichoderma* isolates

Molecular identification of 19 Trichoderma isolates was conducted using DNA sequencing of the internal transcribed regions (ITS1 and ITS4) and a segment of the tef-1α gene. This approach confirmed species identity, complementing earlier morphological assessments. Amplicon sequencing revealed consistent fragment sizes of 650 bp for ITS and 600 bp for tef-1α across all isolates. Sequence analysis and BLAST search confirmed species classification with five isolates identified as *Trichoderma koningiopsis* (TR41, TR28, TR12, TR28, and TR30), six as *Trichoderma brevicompactum* (TR11, TR51, TR4, TR1, TR3, and TR21), two as *Trichoderma caribbaeum* var. *caribbaeum* (TR8 and TR40), one as *Trichoderma atroviride* (TR10), three as *Trichoderma asperellum* (TR44, TR53, and TR20), two as *Trichoderma lixii* (TR17 and TR25), and one as *Trichoderma erinaceum* (TR14). Accession numbers were obtained for submitted gene sequences in the NCBI GenBank database. Geographical coordinates and other relevant data are provided (Table 1). The outgroup species were selected to illustrate phylogenetic relationships, *Mucor circinelloides* (JN205961) for ITS and *Mucor rongii* (MT815277) for tef-1α were chosen as an outgroup.

### Phylogenetic relationship based on ITS rRNA and tef-1α gene sequences

**Figure 2:**
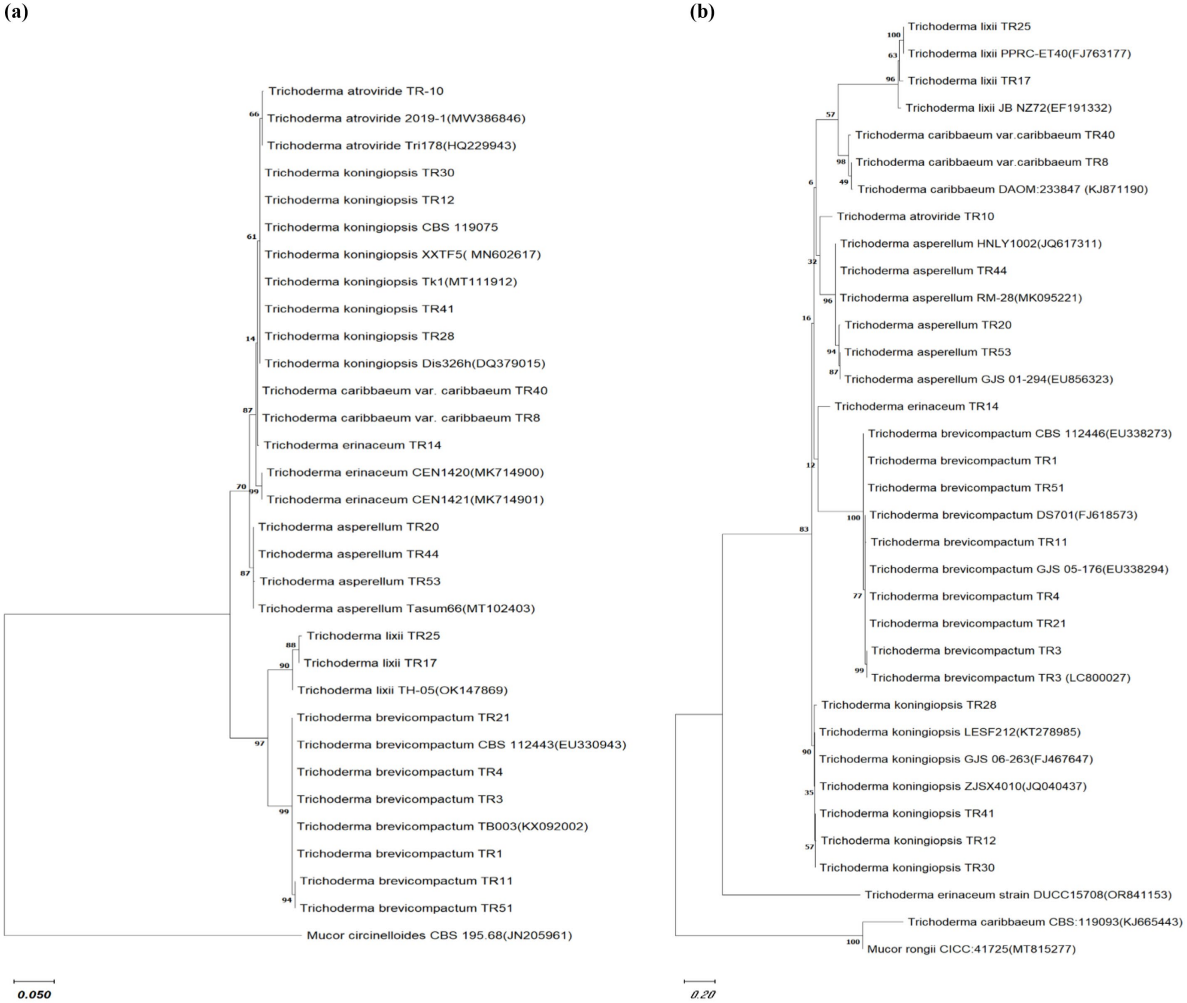
Phylogenetic relationship among *Trichoderma* isolates. Cladogram illustrating the phylogenetic relationships among *Trichoderma* isolates, constructed using the maximum likelihood method based on **(A)** ITS sequences and **(B)** Tef gene sequences. Bootstrap replication was set to 1,000. The comparison utilized sequences obtained from NCBI GenBank with the accession numbers listed in the manuscript. *Mucor circinelloides* CBS 195.68 (JN205961) and *Mucor rongii* CICC:41725(MT815277) were selected as an outgroup for ITS and Tef gene phylogenetic tree, respectively. The scale bar indicates 0.1 and 0.2 substitutions per site for ITS and Tef gene phylogenetic tree, respectively.

Phylogenetic analysis was performed on 19 Trichoderma isolates (TR41, TR8, TR40, TR28, TR25, TR12, TR10, TR30, TR3, TR1, TR17, Tr14, TR20, TR44, TR53, TR11, and TR51) using ITS1, ITS4, and tef-1α sequences. The analysis confirmed their classification within the *Trichoderma* genus. The bootstrap analysis assessed nodal robustness, with numerical values indicating bootstrap support. Based on these values, the 19 *Trichoderma* spp. isolates were categorized into two distinct clades, designated as clade A and clade B, using ITS1 and ITS4 gene sequences, respectively. The *T*. *atroviride* isolate TR10, clustered with *T*. *atroviride* 2019-1 (MW386846) and Tri178 (HQ229943) with a bootstrap value of 66%. The isolates of *T*. *koningiopsis* TR12, TR41, and TR28 clustered with *T*. *koningiopsis*_XXTF5 (MN602617), Tk1 (MT111912), and Dis326h (DQ379015), respectively, with a bootstrap value 63%, whereas *T. caribbaeum* var. *caribbaeum* TR8 clustered with *T. caribbaeum* var. *caribbaeum*_TR10 with a bootstrap value 87%. *T. erinaceum* TR14 clustered with *T. erinaceum* (MK714900 and MK714901) with a bootstrap value of 99%. *T*. *asperellum* (TR44, TR20, and TR53) formed as a distinct clade and clustered with *T. asperellum* (MT102403) at a bootstrap value of 87%. The separated clade B formed two subclades where subclade B1 consists of *T. lixii* (TR25 and TR17) clustered with *T. lixii* (OK147869) and subclade B2 consists of *T. brevicompactum* (TR21, TR4, TR3, and TR1) clustered with *T. brevicompactum* (CBS112443 and TB003) and the outgroup were *M. circinelloides*, respectively (Figure 2A). The bootstrap values indicated the division of the 19 *Trichoderma* spp. isolates into two distinct clades based on tef-1α gene sequences. Clade A consists of four subclades, subclade A1 consists of two subclades, one subclade consists of *T. lixii* (TR25 and TR17) clustered with *T. lixii* (J763177 and EF191332) with a bootstrap value of 100%, and the other subclade consists of *T. caribbaeum* var. *caribbaeum* isolates (TR40 and TR8) clustered with *T. caribbaeum* (KJ871190) with a bootstrap value of 98%. The subclade A2 consists of two clades where *T. atroviride* formed a separate clade and *T. asperellum* (TR44, TR20, and TR53) clustered as a distinct clade with *T. asperellum* (MK095221, JKQ617311, and EU856323) with a bootstrap value of 96%. The subclades A3 consists of two subclades *T*. *erinaceum* isolates TR14 formed a distinct clade, whereas the other subclades consist of *T. brevicompactum* TR1, TR51, TR11, TR21, TR4, and TR3 clustered with *T*. *brevicompactum* (EU338273, FJ618573, and EU338294). The subclades A4 consists of *Trichoderma koningiopsis* (TR28, TR41, TR12, and TR30), clustered with *Trichoderma koningiopsis* (KT278985, FJ467647, and JQ040437) with a bootstrap value of 90%. Clade B consists of *T. erinaceum* (OR841153), which formed a distinct clade, and clade C consists *T. caribbaeum* (KJ665443) with a distinct clade and an outgroup *M. rongii* formed a separate clade (Figure 2B).

### *In vitro* assessment of *Trichoderma* isolates for antagonistic potential

**Figure 3:**
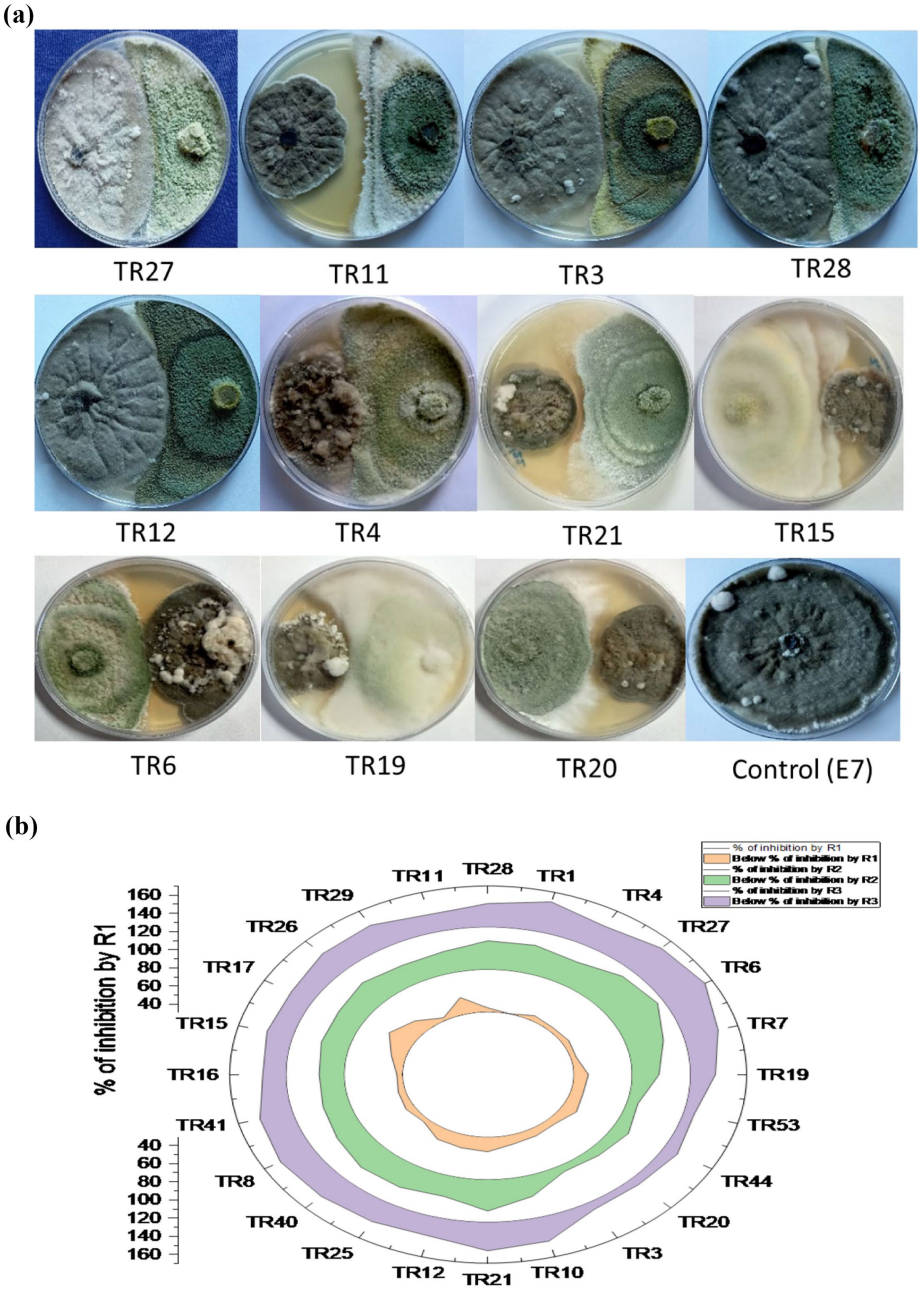
**(A)** Plates illustrate the representative pictures of the antagonistic activity of *Trichoderma* isolates against *Bipolaris maydis* (control) in a dual culture assay under *in vitro* laboratory conditions at 28 ± 2°C. Observations were recorded on the sixth day. **(B)** Stacked radial plot depicts the percentage inhibition among multiple variables in a circular format. Results are expressed as the average of three replications.

The *in vitro* screening of *Trichoderma* isolates revealed a noteworthy reduction in the mycelial growth of *B. maydis*, ranging from 39.52 to 60.00%. Among the isolates, TR11 exhibited the highest inhibition rate at 60.00%, followed by TR21 and TR06, which showed inhibition rates of 58.57 and 58.38%, respectively (Supplementary Table S2 and Figures 3A,B).

### Exploring biochemical defensive enzyme cascade in maize against *Bipolaris maydis* under glasshouse conditions

**Figure 4:**
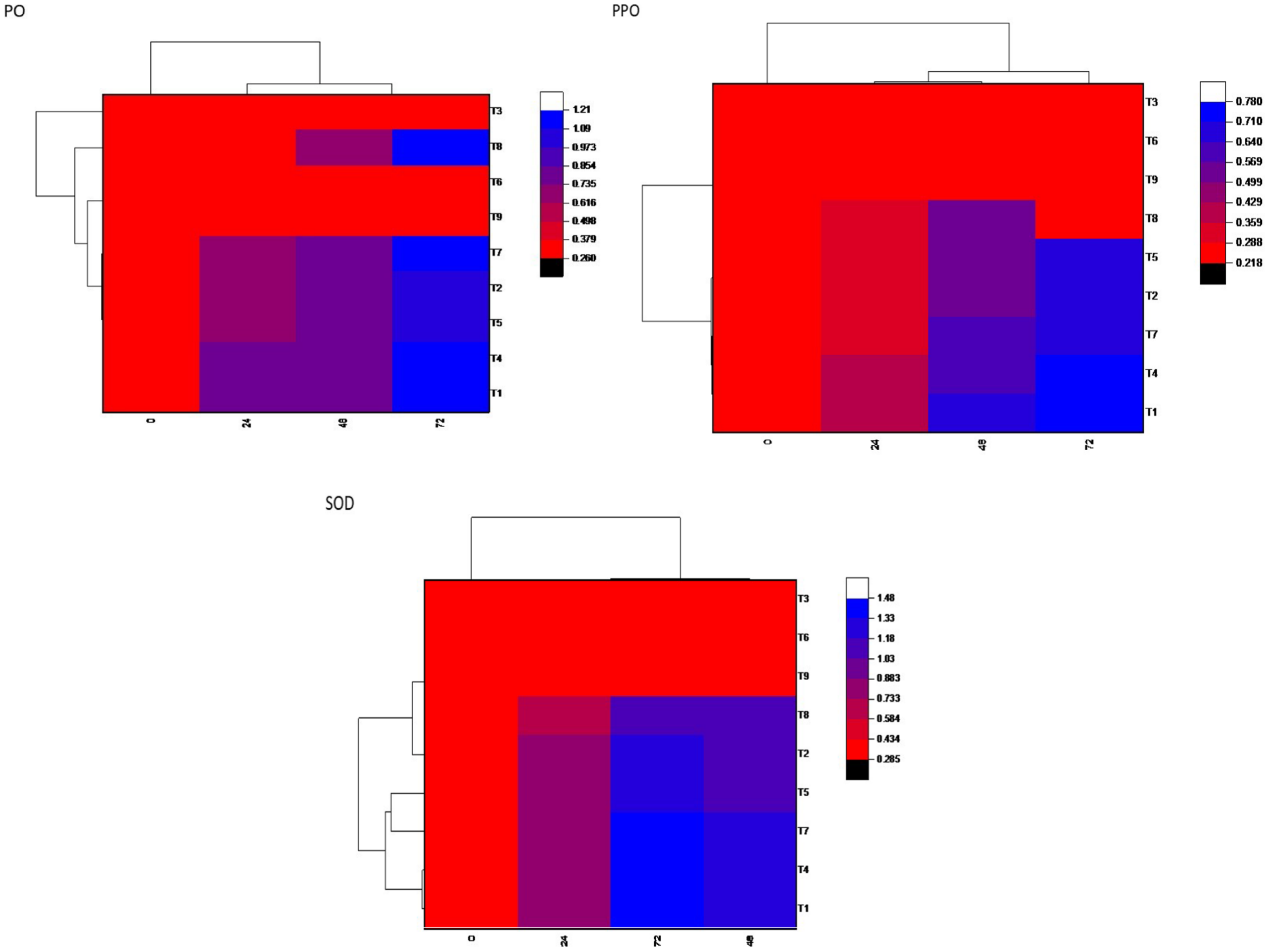
Heatmap showing defensive enzymatic activities in maize plants, postinoculated with *Bipolaris maydis*. Observations were recorded at different time intervals, whereas the *x*-axis shows different time intervals: 0, 24, 48, and 72 h for superoxide dismutase (SOD), polyphenol oxidase (PPO), and peroxidase (PO) activities in maize leaves; and *y*-axis shows different treatments T1: CM212 + E7 + TR11; T2: CM212 + E7; T3: CM212; T4 VL78 + E7 + TR11; T5 VL78 + E7; T6: VL78; T7: Dhiari local +TR11 + E7; T8: Dhiari local+E7; and T9: Dhiari local, where CM212, Dhiari local and VL78 are moderately susceptible cultivar of maize; E7 is the virulent isolate of *Bipolaris maydis*; and TR11 is *Trichoderma* isolate.

The enzymatic activities of superoxide dismutase (SOD), polyphenol oxidase (PPO), and POX were assessed at 0, 24, 48, and 72-h postinoculation with *B. maydis* in 30-day-old maize plants. In all treatments, including seed biopriming with *Trichoderma* isolates, activities of SOD, PPO, and PO were significantly elevated compared to both the pathogen-treated control and the untreated healthy control. Treatment T7 (Dhiari Local+TR11 + E7) exhibited the highest SOD activity, 27.11% higher than the pathogen control at 72 h, followed by treatment T1 (CM212 + TR11 + E7) with a 20.38% increase, where CM212 is a moderately susceptible cultivar of maize, E7 is the virulent isolate of *B. maydis*, and TR11 is the isolate of *Trichoderma*. Treatment T4 showed the highest POX activity, while T1 recorded the highest PPO activity, both exceeding the pathogen control by 13.02 and 12.50%, respectively, at 72 h postinoculation. These enzyme activities remained stable at 0 h, increased at 24 h, and peaked at 72 h postinoculation across all treatments. Conversely, the untreated pathogen control showed no significant increase in enzyme activity (Figure 4).

### Enhancing growth and reducing disease in maize through seed biopriming with *Trichoderma* isolates

**Figure 5:**
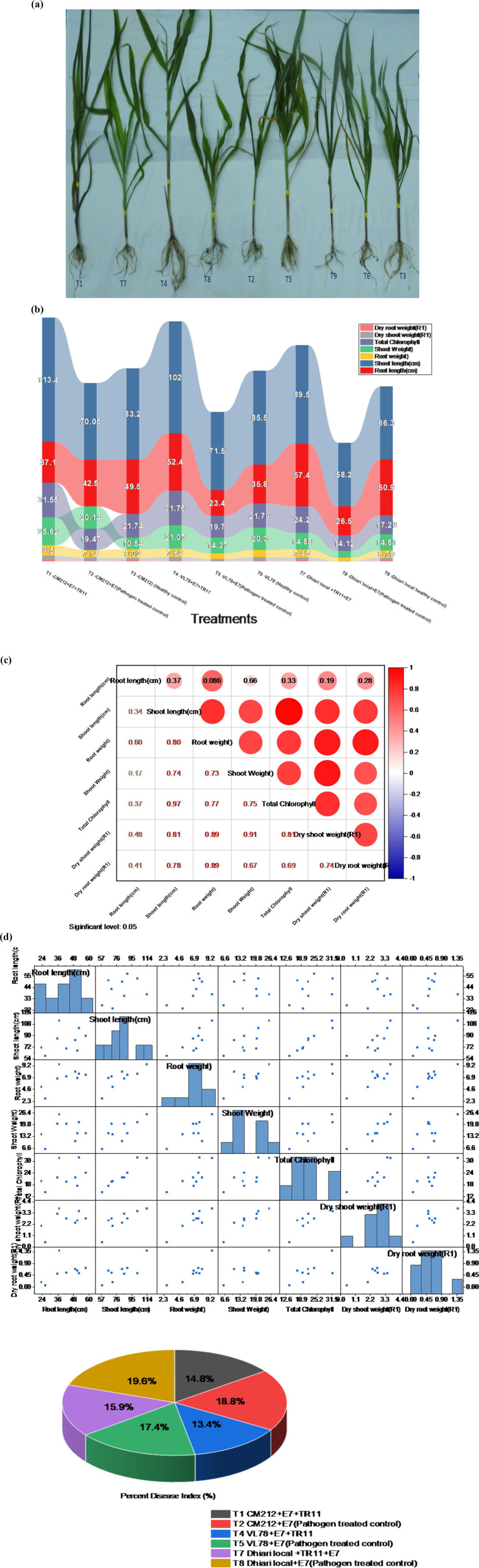
Growth-promotion efficiency of *Trichoderma* strains seed biopriming on various growth parameters of maize crop with different treatments: T1: CM212 + E7 + TR11; T2: CM212 + E7; T3: CM21; T4: VL78 + E7 + TR11; T5: VL78 + E7; T6: VL78; T7: Dhiari local +TR11 + E7; T8: Dhiari local+E7; and T9: Dhiari local, where CM212, Dhiari local, and VL78 are moderately susceptible cultivar of maize; E7 is the virulent isolate of *Bipolaris maydis*; and TR11 is *Trichoderma* isolate. **(A)** Experimental crop after harvest to compare physiological growth, **(B)** growth parameters among various treatments, **(C)** correlation analysis shows all parameters are positively correlated, **(D)** correlation coefficient matrix and associated *p*-values, and **(E)** percent disease index. Results are expressed as the average of three replications.

Significant enhancements in crop physiological growth parameters, such as root length, shoot length, fresh shoot, and root weight, as well as dry shoot and root weight, were observed following seed biopriming treatments compared to the control under controlled glasshouse conditions after a 45-day period post sowing. Among these treatments, treatment T1 (CM212 + E7 + TR11) demonstrated the highest measurements for root length (57.03 cm), shoot length (113.33 cm), fresh shoot weight (25.57 g), root weight (9.15 g), dry shoot weight (3.70 g), dry root weight (1.38 g), and total chlorophyll content (31.12) (Supplementary Table S3). The findings indicate that all *Trichoderma* isolates led to a reduction in the percent disease index (PDI) relative to the pathogen-inoculated control. Treatment T4 (VL78 + E7 + TR11) exhibited the lowest PDI (31.88%), followed by T1 (CM212 + E7 + TR11) (35.22%), which statistically resembled T7 (Dhiari local + TR11 + E7) (37.81%) (Figure 5). Conversely, the highest PDI was recorded in the pathogen-inoculated control across different varieties, with T8 (Dhiari local+E7) (46.60%) showing the highest, followed by T2 (CM212 + E7) (44.74%), at the 45th day after sowing (Supplementary Table S4).

### Induction of lignin deposition in maize roots, induction of callose deposition in maize leaves against pathogen invasion and ROS

Lignin deposition within the cell wall serves as a defensive mechanism against plant pathogen invasion into vascular tissues. Histochemical staining of transverse sections of maize root tissues treated with various methods revealed differences in lignin deposition. The treatments, T1 (CM212 + E7 + TR11), T7 (Dhiari local +TR11 + E7), and T4 (VL78 + E7 + TR11), with *Trichoderma* isolate (TR11), showed the highest lignification in all cultivars compared to the pathogen inoculated and healthy controls. Lignin deposition increased after pathogen inoculation. The strongest increase was observed after treatment with the pathogen only and after combined treatment with TR11 isolate. (Supplementary Figure S1A).

The assessment of reactive oxygen species (ROS) accumulation and callose deposition in maize tissues was conducted to elucidate the innate immune response of plants following inoculation with *Trichoderma*. Notably, ROS, particularly hydrogen peroxide (H_2_O_2_), exhibited marked accumulation in maize leaves 48 h postpathogen inoculation, as evidenced by *in situ* DAB staining. The *Trichoderma*-treated plants also demonstrated induction of H_2_O_2_ accumulation in leaves (Supplementary Figure S1B). Moreover, the analysis of callose deposition, an additional marker of plant innate immune response, in maize leaf tissue after 48 h of treatment, demonstrated a notable rise in both the quantity and area percentage of callose deposition compared to mock controls (Supplementary Figure S1C).

### Analysis of secondary metabolites produced by *Trichoderma* spp. under *in vitro* conditions

To identify metabolites that might be involved in growth promotion and biocontrol effects we further analyzed *T. brevicompactum* TR11 isolate by GC–MS as it was the most promising strain. A total of 88 compounds were identified from the GC–MS analysis. Results of the GC–MS analysis (Supplementary Figure S2 and Table 2), indicated that the main components of *Trichoderma* brevicompactum TR11 filtrate were octadecanoic acid (peak area: 16.27%), palmitic acid-TMS (peak area: 9.44%), phosphoric acid-3TMS (peak area: 7.35%) galactopyranose, 5TMS (peak area: 6.52%), 5-(4-nitrophenoxymethyl) (peak area: 5.43%), furane-2-carboxaldehyde (peak area: 5.43%), stearic acid-TMS (peak area: 4.05%), laevulic acid – TMS (peak area: 3.48%), sucrose, 8TMS(peak area: 3.18%) D-fructose, 6-*O*-[2,3,4,6-tetrakis-*O*-(trimethylsilyl)-*α*-d-glucopyranosyl]-1,3,4,5-tetrakis-*O-*(trimethylsilyl) (peak area: 2.83%), linoleic acid trimethylsilyl ester (peak area: 2.83%), 2-palmitoylglycerol, 2TMS (peak area: 2.53%), 3-methyl-2-furoic acid, TMS (peak area: 2.12%),whereas, other components were present in low percentages; hexadecanoic acid, methyl ester (peak area: 1.39%), d-(−)-fructofuranose, pentakis(trimethylsilyl) ether (isomer 2) (peak area: 1.38%), butylated hydroxytoluene (peak area: 1.13) elaidic acid-TMS (peak area: 1.56%), *cis*-5,8,11,14,17-eicosapentaenoic acid, *tert*-butyldimethylsilyl ester (peak area: 1.51%), lactose, 8TMS (peak area: 1.21%), butylated hydroxytoluene(peak area: 1.13%). On the contrary, there are many components present in traces (peak area less than 1%).

**Table 2:** Detailing compound analysis data.

S. No.	Compound name	RT	Area	% of compound
1	Benzenamine, 2,5-dimethyl-	3.193	362,48	0.06
2	*N*-Trifluoroacetylmorpholine	3.248	54,455	0.09
3	3,6,9-Trioxaundecanedioic acid, bis(trimethylsilyl) ester	3.967	290,536	0.46
4	Dodecane, 2,6,11-trimethyl-	4.062	130,118	0.21
5	Acetic acid, [(trimethylsilyl)oxy]-, trimethylsilyl ester	4.276	132,476	0.21
6	Alanine, 2TMS	4.827	63,366	0.10
7	Dodecane, 2,6,11-trimethyl-	4.91	35,302	0.06
8	Ethyl(2-hydroxyethyl)carbamic acid, *O*,*O*’-bis(trimethylsilyl)	5.505	466,303	0.74
9	Levulic acid – TMS	5.575	2,208,558	3.48
10	α-Hydroxyisovaleric acid, (2TMS)-	6.229	61,147	0.10
11	Trisiloxane, 1,1,1,5,5,5-hexamethyl-3,3-bis[(trimethylsilyl)oxy]-	6.323	24,358	0.04
12	Furane-2-carboxaldehyde, 5-(4-nitrophenoxymethyl)-	6.751	3,439,276	5.43
13	Pentanoic acid, 4-methyl-2-[(trimethylsilyl)oxy]-, trimethylsilyl ester	8.143	16,889	0.03
14	2-Acetylamino-2-methyl-1-propanol, trimethylsilyl ether	8.565	100,668	0.16
15	RT:9.088	9.475	102,771	0.16
16	Phosphoric acid-3TMS	9.326	4,660,031	7.35
17	Hexadecane-1,2-diol, bis(trimethylsilyl) ether	9.59	16,314	0.03
18	Niacin, TMS	9.947	33,460	0.05
19	Phenylacetic acid, monoTMS	10.051	303,526	0.48
20	3-Methyl-2-furoic acid, TMS	10.561	1,346,054	2.12
21	Uracil, 2TMS	11.236	54,149	0.09
22	3,5-Dimethoxymandelic acid, di-TM	11.608	39,513	0.06
23	Fumaric acid, bis-TMS	11.725	63,769	0.10
24	2-Amino-2-(hydroxymethyl)propane-1,3-diol, *N*-acetyl-, tris(trimethylsilyl) ether	11.942	70,679	0.11
25	Tetradecane	13.286	38,264	0.06
26	Glutaric acid-2TMS	13.417	84,494	0.13
27	Eicosane	15.951	188,097	0.30
28	Butanedioic acid, [(trimethylsilyl)oxy]-, bis(trimethylsilyl) ester	15.977	178,561	0.28
29	Butylated hydroxytoluene	16.4	718,658	1.13
30	2-Aminobenzoxazole, 2TMS	17.339	209,419	0.33
31	3-Phenyllactic acid, 2TMS	18.622	16,709	0.03
32	2-Amino-2-(hydroxymethyl)propane-1,3-diol, *N*-acetyl-, tris(trimethylsilyl) ether	19.106	140,325	0.22
33	Hexadecane	19.264	43,029	0.07
34	Cyclooctasiloxane, hexadecamethyl-	20.198	68,688	0.11
35	Tartaric acid-4TMS	20.355	55,492	0.09
36	3,5-Di-*t*-butyl-4-methoxy-1,4-dihydrobenzaldehyde	21.499	302,202	0.48
37	Eicosane	22.039	318,666	0.50
38	Phosphorin, 2,4,6-tris(1,1-dimethylethyl)-	22.819	249,523	0.39
39	Ribose-4TMS	23.068	98,130	0.15
40	Eicosane	23.231	160,961	0.25
41	Cyclononasiloxane, octadecamethyl-	24.371	138,897	0.22
42	Fructose-5TMS	24.849	630,750	1.00
43	d-(−)-Fructofuranose, pentakis(trimethylsilyl) ether (isomer 2)	25.067	876,181	1.38
44	Hexadecyl isopropyl ether	25.665	44,500	0.07
45	β-d-Galactofuranose, 1,2,3,5,6-pentakis-*O*-(trimethylsilyl)-	25.942	1,021,065	1.61
46	D-Xylopyranose, 1,2,3,4-tetrakis-O-(trimethylsilyl)-	26.26	99,809	0.16
47	Galactopyranose, 5TMS	27.076	2,688,256	4.24
48	Eicosane	27.536	341,517	0.54
49	Hexadecanoic acid, methyl ester	27.921	881,521	1.39
50	Cyclononasiloxane, octadecamethyl-	28.081	174,837	0.28
51	n-Pentadecanoic acid	28.446	157,927	0.25
52	Eicosane	28.583	103,152	0.16
53	Galactopyranose, 5TMS	29.371	4,133,277	6.52
54	Palmitic acid-TMS	30.896	5,983,399	9.44
55	Cyclooctasiloxane, hexadecamethyl-	31.475	128,950	0.20
56	Linoleic acid, methyl ester	31.868	463,054	0.73
57	Elaidic acid, methyl ester	32.029	89,988	0.14
58	Methyl stearate	32.657	255,866	0.40
59	Tetrapentacontane	32.508	339,509	0.54
60	Margaric acid-TMS	33.041	93,640	0.15
61	Tetrapentacontane	33.454	105,928	0.17
62	Nonadecanamide	33.866	176,443	0.28
63	Linoleic acid trimethylsilyl ester	34.523	1,791,127	2.83
64	Elaidic acid-TMS	34.646	990,576	1.56
65	cis-5,8,11,14,17-Eicosapentaenoic acid, tert-butyldimethylsilyl ester	35.157	957,151	1.51
66	Stearic acid-TMS	35.255	2,565,588	4.05
67	β-l-Galactopyranose, 6-deoxy-1,2,3,4-tetrakis-*O*-(trimethylsilyl)	36.603	67,454	0.11
68	Tetrapentacontane	37.034	271,038	0.43
69	Cyclononasiloxane, octadecamethyl-	37.403	147,322	0.23
70	Myristic acid, 2,3-bis(trimethylsiloxy)propyl ester	38.39	696,415	1.10
71	Cyclononasiloxane, octadecamethyl-	40.09	173,936	0.27
72	2-Palmitoylglycerol, 2TMS	41.546	1,606,078	2.53
73	Cyclononasiloxane, octadecamethyl-	42.627	1,96,162	0.31
74	Lactose, 8TMS	42.766	765,944	1.21
75	Sucrose, 8TMS	42.98	2,016,418	3.18
76	d-Fructose, 6-*O*-[2,3,4,6-tetrakis-*O*-(trimethylsilyl)-α-d-glucopyranosyl]-1,3,4,5-tetrakis-*O*-(trimethylsilyl)-	43.338	1,790,957	2.83
77	d-(−)-Ribofuranose, tetrakis(trimethylsilyl) ether (isomer 2)	43.592	751,036	1.19
78	Heptadecanoic acid, glycerine-(1)-monoester, bis-*O*-trimethylsilyl-	43.895	100,435	0.16
79	Trehalose-8TMS	44.833	473,701	0.75
80	2-Monostearin, 2TMS	45.038	918,991	1.45
81	3-α-Mannobiose, octakis(trimethylsilyl) ether (isomer 2)	45.513	128,986	0.20
82	Per-*O*-trimethylsilyl-(3-*O*-α-d-mannopyranosyl-4-*O*-β-d-glucopyranosyl-d-glucitol)	45.513	128,986	0.20
83	Glycerol monostearate, 2TMS	45.724	10,311,392	16.27
84	Maltose, 8TMS	47.626	360,735	0.57
85	Maltose, octakis(trimethylsilyl) ether (isomer 1)	47.931	255,695	0.40
86	2,3-Dihydroxypropyl icosanoate, 2TMS	48.882	131,999	0.21
87	Cyclononasiloxane, octadecamethyl-	51.602	81,090	0.13
88	3,5-Cyclo-6,8(14),22-ergostatriene	53.616	148,948	0.24

This includes secondary metabolite compound names, retention times (RT), peak areas (area), and the percentage of each compound in the total composition (% of compound).

## Discussion

In this study, *Trichoderma* strains were isolated and characterized from the Western Ghats of India, reflecting *Trichoderma* as a key mycobiome component in varied ecosystems, such as forests, supported by the studies by Kamala et al. (2015) and Ghorbanpour et al. (2018). In our study, a multigene approach involving the tef-1α, ITS1, and ITS4 gene sequences was used to accurately classify 19 *Trichoderma* isolates into seven distinct species: *T*. *koningiopsis*, *T*. *brevicompactum*, *T*. *caribbaeum* var. *caribbaeum*, *T*. *lixii*, *T*. *asperellum*, *T*. *atroviride*, and *T*. *erinaceum*. Similarly, Zheng et al. (2021) used tef-1α and RNA polymerase II second largest subunit (rpb2) sequences to identify *Trichoderma* isolates, including species such as *T. achlamydosporum* and *T. aquatica*.

*Trichoderma* spp. exhibit strong antagonistic potential through mycoparasitism, competition, antibiosis and induce systemic resistance in plants, which helps reduce the incidence and severity of diseases caused by phytopathogens (Atanasova et al., 2013; Vargas et al., 2014; Cherkupally et al., 2017). According to the *in vitro* results, the *T*. *brevicompactum* TR11 isolates exhibited significant inhibitory potential against the *B. maydis* E7 isolates, restricting its mycelial growth by up to 60% compared to other isolates in the dual culture assay. Numerous studies have supported the antagonistic potential of certain *Trichoderma* species, against *Exserohilum turcicum*, *Bipolaris oryzae*, and *Colletotrichum graminicola* (Ma et al., 2023; Khalili et al., 2012; Manzar et al., 2021). The findings from the glasshouse experiments highlight the antagonistic capabilities of *Trichoderma* isolates against *B. maydis*, in maize plants. Treatment T1 (CM212 + E7 + TR11), which comprised the moderately susceptible maize variety CM212, the virulent strain E7, and the *Trichoderma* isolate TR11, exhibited significant improvements in several plant growth parameters, such as root and shoot lengths, fresh and dry weights of shoots, as well as a reduction in disease severity compared to the pathogen-inoculated control under glasshouse conditions. In a previous study, seeds of *Celosia cristata* treated with *Trichoderma harzianum* Th62 also showed significantly increased plant height, diameter of the stem, number of branches, dry weight of root, stem, leaf, and flower and enhanced resistance against *Alternaria alternata*, *Rhizoctonia solani*, *Cystospora chrysosperma, Sclerotinia sclerotiorum*, and *Fusarium oxysporum* (Hou et al., 2021).

In our study treatment T1, which combined the moderately susceptible maize variety CM212, the *B. maydis* virulent strain E7, and the *Trichoderma* isolate TR11, showed a marked increase in the activities of defense enzymes such as POX, polyphenol oxidase, and superoxide dismutase compared to pathogen-treated control and the untreated, unchallenged healthy control. This increased defense enzymatic activity is vital for reinforcing the plant defense mechanisms and, as a result, slows the progression of the disease. Our findings align with existing literature that highlights the importance of enzymes such as polyphenol oxidase and POX in biological control and plant disease resistance (Zhang et al., 2008; Petrov and Van Breusegem, 2012; Singh and Singh, 2012; Kashyap et al., 2021). In our experiments, maize plants treated with *Trichoderma* isolates TR11 displayed significant callose deposition and lignification levels, which contributed to their resistance against MLB. This is consistent with the findings that *Trichoderma* spp. treated plants exhibited increased activities of various defense enzymes, including glucanase, POX, and polyphenol oxidase, alongside enhanced cell wall lignification and callose deposition compared to untreated controls (Singh et al., 2019; Keswani et al., 2019; Keswani et al., 2014; Siddaiah et al., 2017; Ben Amira et al., 2018; Kashyap et al., 2022). The TR11 isolate exhibited strong biocontrol potential, demonstrating both growth promotion and antagonism through experiments on antimicrobial activity, *in vitro* antibiosis, pot trials for plant growth promotion, and induction of systemic resistance. A total of 88 compounds were detected in the ethyl acetate extracts. The primary constituents include fatty acids, esters, aldehydes, hydrazides, pyrazines, imidazoles, triazines, and fatty amides. The primary active ingredient in our investigations *T. brevicompactum* TR11 isolate acetonic extract was glycerol monostearate 2TMS, followed by palmitic acid-TMS and phosphoric acid-3TMS in the *Trichoderma* TR11 filtrate. Similar findings were found in *Trichoderma* species, such as *T. harzianum* and *Trichoderma viride* produce secondary metabolites such as butanoic acid, propanoic acid, and hexadecanoic acid, which inhibit the growth of phytopathogenic fungi, such as *Colletotrichum lagenarum*, *S. sclerotiorum*, and *Cercospora beticola* (Agoramoorthy et al., 2007; Liu et al., 2008; Altieri et al., 2007; Avis et al., 2000; Pohl et al., 2011). The fatty acids such as pentadecanoic acid, butanedioic acid, dodecanoic acid, heptadecanoic acid, palmitic acid, and octadecenoic acid exhibit antifungal and antibacterial properties against various plant pathogens.

## Conclusion

Exploring the biodiversity hotspot of the Western Ghats of India, *Trichoderma* isolates were meticulously screened, revealing 7% with robust biocontrol potential and growth-promoting activities. Notably, three novel *Trichoderma* isolates—*T. caribbaeum* var. *caribbaeum*, *T. lixii*, and *T. brevicompactum*—were reported for the first time from this region. Molecular characterization, based on ITS-rRNA and tef-1*α* gene sequencing, identified major isolates, enhancing our understanding of genetic diversity. *In vitro* screening demonstrated substantial mycelial growth inhibition by 24 *Trichoderma* spp. isolates against *B. maydis*, underscoring their biocontrol efficacy. The biochemical defense mechanisms in maize, including elevated enzymatic activities and seed biopriming with Trichoderma isolates TR11, showcased promising disease reduction and growth promotion attributes. Induction of lignin and callose depositions in maize roots and leaves postpathogen invasion highlighted intricate defense responses. This study provides a comprehensive insight into the multifaceted roles of *Trichoderma* spp. in boosting plant health and combating phytopathogens, emphasizing their potential in sustainable agriculture and paving the way for the future research endeavors in crop protection and productivity enhancement.

## Data Availability

The datasets presented in this study can be found in online repositories. The names of the repository/repositories and accession number(s) can be found at: NCBI database.

## References

[ref1] Aggarwal S. K. Hooda K. S. Kaur H. Gogoi R. Chauhan P. Bagaria P. K.. (2024). Comparative evaluation of management modules against Maydis leaf blight disease in maize (*Zea mays*). Eur. J. Plant Pathol. 168, 485–495. doi: 10.1007/s10658-023-02777-x

[ref2] Agoramoorthy G. Chandrasekaran M. Venkatesalu V. Hsu M. (2007). Antibacterial and antifungal activities of fatty acid methyl esters of the blind-your-eye mangrove from India. Braz. J. Microbiol. 38, 739–742. doi: 10.1590/S1517-83822007000400028

[ref3] Altieri C. Cardillo D. Bevilacqua A. Sinigaglia M. (2007). Inhibition of aspergillus spp. and Penicillium spp. by fatty acids and their monoglycerides. J. Food Prot. 70, 1206–1212. doi: 10.4315/0362-028X-70.5.1206, PMID: 17536681

[ref4] Atanasova L. Le Crom S. Gruber S. Coulpier F. Seidl-Seiboth V. Kubicek C. P.. (2013). Comparative transcriptomics reveals different strategies of Trichoderma mycoparasitism. BMC Genomics 14:121. doi: 10.1186/1471-2164-14-121, PMID: 23432824 PMC3599271

[ref5] Avis T. Boulanger R. Bélanger R. (2000). Synthesis and biological characterization of (Z)-9-heptadecenoic and (Z)-6-methyl-9-heptadecenoic acids: fatty acids with antibiotic activity produced by Pseudozyma focculosa. J. Chem. Ecol. 26, 987–1000. doi: 10.1023/A:1005464326573

[ref6] Barnett H. L. Hunter B. B. (1972). Illustrated Genera of Imperfect Fungi. 3rd Edition. Minneapolis: Burgess Publishing Co., 241 p.

[ref7] Beauchamp C. Fridovich I. (1971). Superoxide dismutase: improved assays and an assay applicable to acrylamide gels. Anal. Biochem. 44, 276–287. doi: 10.1016/0003-2697(71)90370-8, PMID: 4943714

[ref8] Ben Amira M. Mom R. Lopez D. Chaar H. Khouaja A. Pujade-Renaud V.. (2018). MIP diversity from Trichoderma: structural considerations and transcriptional modulation during mycoparasitic association with Fusarium solani olive trees. PLoS One 13:e0193760. doi: 10.1371/journal.pone.0193760, PMID: 29543834 PMC5854309

[ref9] Bindschedler L. V. Dewdney J. Blee K. A. Stone J. M. Asai T. Plotnikov J.. (2006). Peroxidase-dependent apoplastic oxidative burst in Arabidopsis required for pathogen resistance. Plant J. 47, 851–863. doi: 10.1111/j.1365-313X.2006.02837.x, PMID: 16889645 PMC3233234

[ref10] Bissett J. Szakacs G. Nolan C. A. Druzhinina I. Gradinger C. Kubicek C. P. (2003). New species of Trichoderma from Asia. Can. J. Bot. 81, 570–586. doi: 10.1139/b03-051

[ref12] Cherkupally R. Amballa H. Reddy B. N. (2017). In vitro screening for enzymatic activity of Trichoderma species for biocontrol potential. Ann. Plant Sci. 6, 1784–1789. doi: 10.21746/aps.2017.6.11.11

[ref13] Chugh V. Kaur N. Gupta A. K. (2011). Evaluation of oxidative stress tolerance in maize (*Zea mays* L.) seedlings in response to drought. Indian J. Biochem. Biophys. 48, 47–53, PMID: 21469602

[ref14] Crutcher F. K. Parich A. Schuhmacher R. Mukherjee P. K. Zeilinger S. Kenerley C. M. (2013). A putative terpene cyclase, vir4, is responsible for the biosynthesis of volatile terpene compounds in the biocontrol fungus Trichoderma virens. Fungal Genet. Biol. 56, 67–77. doi: 10.1016/j.fgb.2013.05.003, PMID: 23707931

[ref15] Daudi A. O'Brien J. A. (2012). Detection of hydrogen peroxide by DAB staining in Arabidopsis leaves. Bio Protocol 2. doi: 10.21769/BioProtoc.263 PMC4932902 27390754

[ref16] Druzhinina I. S. Kopchinskiy A. G. Komoń M. Bissett J. Szakacs G. Kubicek C. P. (2005). An oligonucleotide barcode for species identification in Trichoderma and Hypocrea. Fungal Genet. Biol. 42, 813–828. doi: 10.1016/j.fgb.2005.06.007, PMID: 16154784

[ref17] Esparza-Reynoso S. Ruíz-Herrera L. Pelagio-Flores R. Macías-Rodríguez L. I. Martínez-Trujillo M. López-Coria M.. (2021). Trichoderma atroviride-emitted volatiles improve growth of Arabidopsis seedlings through modulation of sucrose transport and metabolism. Plant Cell Environ. 44, 1961–1976. doi: 10.1111/pce.14014, PMID: 33529396

[ref18] Fan L. Linker R. Gepstein S. Tanimoto E. Yamamoto R. Neumann P. M. (2006). Progressive inhibition by water deficit of cell wall extensibility and growth along the elongation zone of maize roots is related to increased lignin metabolism and progressive stelar accumulation of wall phenolics. Plant Physiol. 140, 603–612. doi: 10.1104/pp.105.073130, PMID: 16384904 PMC1361327

[ref9004] Gherbawy Y. Druzhinina I. Shaban G. M. Wuczkowsky M. Yaser M. A. Prillinger H. J.. (2004). Trichoderma populations from alkaline agricultural soil in the Nile valley,Egypt,consist of only two species. Mycological Progress. 3:211218.

[ref9002] Ghorbanpour A. Salimi A. Ghanbary M. A. T. Pirdashti H. Dehestani A. (2018). The effect of Trichoderma harzianum in mitigating low temperature stress in tomato (Solanum lycopersicum L.) plants. Sci. Hortic. 230, 134–141.

[ref19] Gogoi R. Singh S. Singh P. K. Kulanthaivel S. Rai S. N. (2014). Genetic variability in the isolates of Bipolaris maydis causing maydis leaf blight of maize. Afr. J. Agric. Res. 9, 1906–1913.

[ref9003] Guo Z. Ou W. Lu S. Zhong Q. (2006). Differential responses of antioxidative system to chilling and drought in four rice cultivars differing in sensitivity. Plant Physiology and Biochemistry, 44, 828–836. doi: 10.1016/j.plaphy.2006.10.024 17098438

[ref9031] Hall T. A. (1999). BioEdit: A user-friendly biological sequence alignment editor and analysis program for Windows 95/98/NT. Nucleic Acids Symp. Ser., 41, 95–98.

[ref9001] Hammerschmidt R Nuckles EM Kuć J. (1982). Association of enhanced peroxidase activity with induced systemic resistance of cucumber to Colletotrichum lagenarium. Physiol Plant Pathol 20, 73–82. doi: 10.1016/0048-4059(82)90025-X

[ref20] Harman G. E. (2011). Trichoderma—not just for biocontrol anymore. Phytoparasitica 39, 103–108. doi: 10.1007/s12600-011-0151-y

[ref21] Hou X. Y. Wang Y. F. Jiang C. Y. Zhai T. T. Miao R. Deng J. J.. (2021). A native Trichoderma harzianum strain Th62 displays antagonistic activities against phytopathogenic fungi and promotes the growth of *Celosia cristata*. Hortic. Environ. Biotechnol. 62, 169–179. doi: 10.1007/s13580-020-00307-w

[ref22] Hoyos-Carvajal L. Orduz S. Bissett J. (2009). Genetic and metabolic biodiversity of Trichoderma from Colombia and adjacent neotropic regions. Fungal Genet. Biol. 46, 615–631. doi: 10.1016/j.fgb.2009.04.006, PMID: 19439189

[ref23] Jaklitsch W. M. (2011). European species of Hypocrea part II: species with hyaline ascospores. Fungal Divers. 48, 1–250. doi: 10.1007/s13225-011-0088-y, PMID: 21994484 PMC3189789

[ref24] Jie C. Kai D. Yong Dong G. Qia L. I. Y. (2014). “Mechanism and application of *Trichoderma* spp” in Biological control of corn diseases Mycosystema. 33:1154–1167. doi: 10.13346/j.mycosystema.140501

[ref25] Kamala T. Devi S. I. Sharma K. C. Kennedy K. (2015). Phylogeny and taxonomical investigation of Trichoderma spp. from Indian region of Indo-Burma biodiversity hot spot region with special reference to Manipur. Biomed. Res. Int. 2015:285261. doi: 10.1155/2015/285261, PMID: 25699268 PMC4324893

[ref26] Kashyap A. S. Manzar N. Maurya A. Mishra D. D. Singh R. P. Sharma P. K. (2023). Development of diagnostic markers and applied for genetic diversity study and population structure of Bipolaris sorokiniana associated with leaf blight complex of wheat. J. Fungi 9:153. doi: 10.3390/jof9020153, PMID: 36836268 PMC9968152

[ref27] Kashyap A. S. Manzar N. Nebapure S. M. Rajawat M. V. S. Deo M. M. Singh J. P.. (2022). Unraveling microbial volatile elicitors using a transparent methodology for induction of systemic resistance and regulation of antioxidant genes at expression levels in chili against bacterial wilt disease. Antioxidants 11:404. doi: 10.3390/antiox11020404, PMID: 35204287 PMC8869530

[ref28] Kashyap A. S. Manzar N. Rajawat M. V. S. Kesharwani A. K. Singh R. P. Dubey S. C.. (2021). Screening and biocontrol potential of Rhizobacteria native to Gangetic Plains and hilly regions to induce systemic resistance and promote plant growth in chilli against bacterial wilt disease. Plan. Theory 10:2125. doi: 10.3390/plants10102125, PMID: 34685934 PMC8541367

[ref29] Keswani C. Dilnashin H. Birla H. Singh S. (2019). Unravelling efficient applications of agriculturally important microorganisms for alleviation of induced inter-cellular oxidative stress in crops. Acta Agric. Slovenica 114:121. doi: 10.14720/aas.2019.114.1.14

[ref30] Keswani C. Mishra S. Sarma B. K. Singh S. P. Singh H. B. (2014). Unraveling the efficient applications of secondary metabolites of various Trichoderma spp. Appl. Microbiol. Biotechnol. 98, 533–544. doi: 10.1007/s00253-013-5344-5, PMID: 24276619

[ref31] Khalili E. Sadravi M. Naeimi S. Khosravi V. (2012). Biological control of rice brown spot with native isolates of three Trichoderma species. Braz. J. Microbiol. 43, 297–305. doi: 10.1590/S1517-83822012000100035, PMID: 24031832 PMC3768959

[ref32] Kraus G. F. Druzhinina I. Gams W. Bissett J. Zafari D. Szakacs G.. (2004). Trichoderma brevicompactum sp. nov. Mycologia 96, 1059–1073. doi: 10.2307/3762089, PMID: 21148926

[ref33] Kubicek C. P. Komon-Zelazowska M. Druzhinina I. S. (2008). Fungal genus Hypocrea/Trichoderma: from barcodes to biodiversity. J Zhejiang Univ Sci B 9, 753–763. doi: 10.1631/jzus.b0860015, PMID: 18837102 PMC2565738

[ref34] Kullnig C. Szakacs G. Kubicek C. P. (2000). Molecular identification of Trichoderma species from Russia, Siberia and the Himalaya. Mycol. Res. 104, 1117–1125. doi: 10.1017/s0953756200002604

[ref36] Lewis J. A. Papavizas G. C. Lumsden R. D. (1991). A new formulation system for the application of biocontrol fungi to soil. Biocontrol Sci. Tech. 1, 59–69. doi: 10.1080/09583159109355186

[ref37] Li Q. R. Tan P. Jiang Y. L. Hyde K. D. Mckenzie E. H. C. Bahkali A. H.. (2013). A novel Trichoderma species isolated from soil in Guizhou, T. guizhouense. Mycol. Progr. 12, 167–172. doi: 10.1007/s11557-012-0821-2

[ref38] Liu S. Ruan W. Li J. Xu H. Wang J. Gao Y.. (2008). Biological control of phytopathogenic fungi by fatty acids. Mycopathologia 166, 93–102. doi: 10.1007/s11046-008-9124-1 18443921

[ref40] Manzar N. Kashyap A. S. Goutam R. S. Rajawat M. V. S. Sharma P. K. Sharma S. K.. (2022a). Trichoderma: advent of versatile biocontrol agent, its secrets and insights into mechanism of biocontrol potential. Sustain. For. 14:12786. doi: 10.3390/su141912786

[ref41] Manzar N. Kashyap A. S. Maurya A. Rajawat M. V. S. Sharma P. K. Srivastava A. K.. (2022b). Multi-gene phylogenetic approach for identification and diversity analysis of Bipolaris maydis and Curvularia lunata isolates causing foliar blight of *Zea mays*. J. Fungi 8:802. doi: 10.3390/jof8080802, PMID: 36012790 PMC9410300

[ref42] Manzar N. Singh Y. (2020). Integration of seed biopriming, soil, and foliar application of formulations of Trichoderma species for management of anthracnose of sorghum under field conditions. Int. J. Curr. Microbiol. Appl. Sci. 9, 334–344. doi: 10.20546/ijcmas.2020.901.038

[ref43] Manzar N. Singh Y. Kashyap A. S. Sahu P. K. Rajawat S. M. V. Bhowmik A.. (2021). Biocontrol potential of native Trichoderma spp. against anthracnose of great millet (sorghum bicolour L.) from Tarai and hill regions of India. Biol. Control 152:104474. doi: 10.1016/j.biocontrol.2020.104474

[ref9005] Mashima J. Kodama Y. Fujisawa T. Katayama T. Okuda Y. Kaminuma. (2017). DNA Data Bank of Japan. Nucleic Acids Res. 45, D25–D31. doi: 10.1093/nar/gkw1001 27924010 PMC5210514

[ref44] Mayer A. Harel E. Ben-Shaul R. (1966). Assay of catechol oxidase—a critical comparison of methods. Phytochemistry 5, 783–789. doi: 10.1016/S0031-9422(00)83660-2

[ref39] Ma Y. Li Y. Yang S. Li Y. Zhu Z. (2023). Biocontrol potential of Trichoderma asperellum strain 576 against Exserohilum turcicum in *Zea mays*. J. Fungi 9:936. doi: 10.3390/jof9090936, PMID: 37755043 PMC10532967

[ref45] Mehta D. Shah D. (2021). Cyanobacteria and microalgae growing on monuments of UNESCO world heritage site Champaner Pavagadh, India: biofilms and their exopolysaccharide composition. Arch. Microbiol. 203, 3425–3433. doi: 10.1007/s00203-021-02334-2, PMID: 33891130

[ref46] Mokhtar H. Aid D. (2013). Contribution in isolation and identification of some pathogenic fungi from wheat seeds, and evaluation of antagonistic capability of Trichoderma harzianum against those isolated fungi in vitro. Agric. Biol. J. N. Am. 4, 145–154. doi: 10.5251/abjna.2013.4.2.145.154

[ref47] Morton D. T. Stroube N. H. (1955). Antagonistic and stimulatory effects of microorganism upon Sclerotium rolfsii. Phytopathology 45, 419–420.

[ref48] Muimba-Kankolongo A. Bergstrom G. C. (2011). Reduced anthracnose stalk rot in resistant maize is associated with restricted development of Colletotrichum graminicola in pith tissues. J. Phytopathol. 159, 329–341. doi: 10.1111/j.1439-0434.2010.01766.x

[ref49] Nampoothiri K. M. Ramkumar B. Pandey A. (2013). Western Ghats of India: rich source of microbial biodiversity. J. Sci. Indust Res. 72, 617–623.

[ref50] Nieto-Jacobo M. F. Steyaert J. M. Badillo F. B. S. Nguyen D. V. Rostás M. Braithwaite M.. (2017). Environmental growth conditions of Trichoderma spp. affects indole acetic acid derivatives, volatile organic compounds, and plant growth promotion. Front. Plant Sci. 8:102. doi: 10.3389/fpls.2017.00102 28232840 PMC5299017

[ref51] Petrov V. D. Van Breusegem F. (2012). Hydrogen peroxide-a central hub for information flow in plant cells. AoB Plants 2012:pls014. doi: 10.1093/aobpla/pls014, PMID: 22708052 PMC3366437

[ref52] Pohl C. H. Kock J. L. Thibane V. S. (2011). Antifungal free fatty acids: a review. Sci. Against Microb. Pathogens 1, 61–71.

[ref53] Rodriguez M. D. C. H. Evans H. C. Abreu L. M. D. (2021). New species and records of Trichoderma isolated as mycoparasites and endophytes from cultivated and wild coffee in Africa. Sci. Rep. 11, 56–71. 33707461 10.1038/s41598-021-84111-1 PMC7952591

[ref54] Samuels G. J. (2006). Trichoderma: systematics, the sexual state, and ecology. Phytopathology 96, 195–206. doi: 10.1094/phyto-96-0195, PMID: 18943925

[ref9032] Sayers E. W. Bolton E. E. Brister J. R. Canese K. Chan J. Comeau D. C., (2022). Database resources of the National Center for Biotechnology Information. Nucleic Acids Res., 50, D20–D26. doi: 10.1093/nar/gkab1112 34850941 PMC8728269

[ref56] Siddaiah C. N. Satyanarayana N. R. Mudili V. (2017). Elicitation of resistance and associated Defense responses in Trichoderma hamatum induced protection against pearl millet downy mildew pathogen. Nat. Publ. Gr. 7, 1–18. doi: 10.1038/srep43991, PMID: 28322224 PMC5359564

[ref57] Siddiquee S. Cheong B. E. Taslima K. Kausar H. Hasan M. M. (2012). Separation and identification of volatile compounds from liquid cultures of Trichoderma harzianum by GC-MS using three different capillary columns. J. Chromatogr. Sci. 50, 358–367. doi: 10.1093/chromsci/bms012, PMID: 22407347

[ref60] Singh B. N. Singh A. Singh S. P. Singh H. B. (2011). Trichoderma harzianum-mediated reprogramming of oxidative stress response in root apoplast of sunflower enhances defense against Rhizoctonia solani. Eur. J. Plant Pathol. 131, 121–134. doi: 10.1007/s10658-011-9792-4

[ref58] Singh H. B. Keswani C. Reddy M. S. Sansinenea E. García-Estrada C. (2019). Secondary metabolites of plant growth promoting rhizomicroorganisms: discovery and applications: Springer.

[ref59] Singh S. P. Singh H. (2012). Effect of consortium of Trichoderma harzianum isolates on growth attributes and Sclerotinia sclerotiorum rot of brinjal. Veget. Sci. 39, 144–148.

[ref61] Stoppacher N. Kluger B. Zeilinger S. Krska R. Schuhmacher R. (2010). Identification and profiling of volatile metabolites of the biocontrol fungus Trichoderma atroviride by HS-SPME-GC-MS. J. Microbiol. Methods 81, 187–193. doi: 10.1016/j.mimet.2010.03.011, PMID: 20302890

[ref62] Sun R. Liu Z. Fu K. (2012). Trichoderma biodiversity in China. J. Appl. Genet. 53, 343–354. doi: 10.1007/s13353-012-0093-1, PMID: 22528042

[ref9033] Tamura K. Nei M. (1993). Estimation of the number of nucleotide substitutions in the control region of mitochondrial DNA in humans and chimpanzees. Molecular Biology and Evolution, 10, 512–526. doi: 10.1093/oxfordjournals.molbev.a040023 8336541

[ref9034] Tamura K. Stecher G. Kumar S. (2021). MEGA11: Molecular Evolutionary Genetics Analysis version 11. Mol. Biol. Evol., 38, 3022–3027. doi: 10.1093/molbev/msab120 33892491 PMC8233496

[ref63] Thompson J. D. Higgins D. G. Gibson T. J. (1994). Clustal W: improving the sensitivity of progressive multiple sequence alignment through sequence weighting, position-specific gap penalties and weight matrix choice. Nucleic Acids Res. 22, 4673–4680. doi: 10.1093/nar/22.22.4673, PMID: 7984417 PMC308517

[ref64] Thordal-Christensen H. Zhang Z. Wei Y. Collinge D. B. (1997). Subcellular localization of H2O2 in plants. H2O2 accumulation in papillae and hypersensitive response during the barley—powdery mildew interaction. Plant J. 11, 1187–1194. doi: 10.1046/j.1365-313X.1997.11061187.x

[ref65] Trillas M. I. Casanova E. Cotxarrera L. Ordovas J. Borrero C. Aviles M. (2006). 468 composts from agricultural waste and the Trichoderma asperellum strain T-34 469 suppress Rhizoctonia solani in cucumber seedlings. Biol. Control 39, 32–38. doi: 10.1016/j.biocontrol.2006.05.007

[ref66] Vargas W. A. Mukherjee P. K. Laughlin D. Wiest A. Moran-Diez M. E. Kenerley C. M. (2014). Role of gliotoxin in the symbiotic and pathogenic interactions of Trichoderma virens. Microbiology 160, 2319–2330. doi: 10.1099/mic.0.079210-0, PMID: 25082950

[ref67] Vinale F. Manganiello G. Nigro M. Mazzei P. Piccolo A. Pascale A.. (2014). A novel fungal metabolite with beneficial properties for agricultural applications. Molecules 19, 9760–9772. doi: 10.3390/molecules19079760, PMID: 25006784 PMC6271495

[ref68] Wheeler B. E. J. (1969). An introduction to plant diseases. London: John Wiley and Sons, Ltd.

[ref69] Zhang C.-L. Druzhinina I. S. Kubicek C. P. Xu T. (2005). Trichoderma biodiversity in China: evidence for a North to South distribution of species in East Asia. FEMS Microbiol. Lett. 251, 251–257. doi: 10.1016/j.femsle.2005.08.034, PMID: 16165315

[ref70] Zhang X. Liu S. Takano T. (2008). Two cysteine proteinase inhibitors from *Arabidopsis thaliana*, AtCYSa and AtCYSb, increasing the salt, drought, oxidation, and cold tolerance. Plant Mol. Biol. 68, 131–143. doi: 10.1007/s11103-008-9357-x, PMID: 18523728

[ref71] Zheng H. Qiao M. Lv Y. Du X. Zhang K.-Q. Yu Z. (2021). New species of Trichoderma isolated as endophytes and saprobes from Southwest China. J. Fungi 7:467. doi: 10.3390/jof7060467, PMID: 34207925 PMC8230185

[ref72] Zhu M. Tong L. Xu M. Zhong T. (2021). Genetic dissection of maize disease resistance and its applications in molecular breeding. Mol. Breed. 41:32. doi: 10.1007/s11032-021-01219-y, PMID: 37309327 PMC10236108

