# Supplementary material for: Exploring Trichoderma diversity in the Western Ghats of India: phylogenetic analysis, metabolomics insights and biocontrol efficacy against Maydis Leaf Blight disease
Source: Front Microbiol. 2024 Dec 20;15:1493272. doi: 10.3389/fmicb.2024.1493272 (PMC11695376; doi:10.3389/fmicb.2024.1493272)
Supplement: Supplementary file 1 [file Table_1.docx]

Supplementary Table 1: Morphological Traits of Trichoderma Species

| Isolates | Colony  colour | Morphology of Mycelia | Pigmentation |
| --- | --- | --- | --- |
| TR34-TR41 | White to light green | Fluffy mycelia, with rough edges | Cream |
| TR3 | Light Green | Rough and flat edges, concentric ring absent | Yellow |
| TR15-TR8 | White to light green | Fluffy mycelia, rough edges | Cream |
| TR16-TR40 | White to light green | Fluffy mycelia, rough edges | Yellow |
| TR10 | Green | Velvety mycelia, smooth edges | Yellow |
| TR9-TR44 | White to light green | Velvety mycelia, rough edges | Cream |
| TR4 | White to light green | Fluffy mycelia | Cream |
| TR25 | White to light green | Velvety smooth mycelia | Yellow |
| TR19 | White to light green | Fluffy dense mycelia | Yellow |
| TR12 | Green | Velvety smooth mycelia | Yellow |
| TR28 | White to light green | Fluffy growth with rough edges | Yellow |
| TR17 | Yellowish green | Rough edges, fluffy mycelia | Yellow |
| TR6-TR20 | Green mycelia | Smooth mycelia, rough edges | Cream |
| TR30-TR55 | White to green | Fluffy mycelia, smooth edges | Cream |
| TR27-TR53 | Dark green | Rough mycelia with flat edges | Yellow |
| TR14 | Light green | Velvety growth | Yellow |
| TR30 | White to Light green | Fluffy growth with smooth edges | Yellow |
| TR11 | Dark green | Velvety mycelia with smooth edges | Cream |
| TR17-TR21 | Yellowish green | Fluffy mycelia with rough egdes | Yellow |
| TR28-TR51 | Green | Smooth velvety mycelia | Cream |
| TR2 | Dark green | Smooth velvety mycelia | Yellow |
| TR1 | Dark green | Fluffy mycelia with smooth edges | Yellow |
| TR18 | White to light green | F  luffy growth with smooth edges | Cream |
| TR28 |  |  |  |
| TR5 | Greenish | Smooth velvety mycelia | Cream |

| Isolates | % of inhibition |
| --- | --- |
| TR19 | 49.05±0.41g |
| TR7 | 52.38±0.21d |
| TR11 | 60.00±0.34a |
| TR27 | 56.67±0.45b |
| TR4 | 50.95±0.67f |
| TR1 | 53.33±0.24c |
| TR28 | 51.91±0.97e |
| TR06 | 58.38±0.35d |
| TR29 | 49.05±0.68g |
| TR26 | 53.81±0.72c |
| TR17 | 53.33±0.23c |
| TR15 | 50.00±0.24f |
| TR16 | 45.72±0.51h |
| TR41 | 49.52±0.64g |
| TR8 | 51.90±0.95e |
| TR40 | 51.43±0.23e |
| TR25 | 52.86±0.96d |
| TR12 | 50.95±0.45f |
| TR21 | 58.57±0.43b |
| TR10 | 51.43±0.21e |
| TR3 | 39.52±0.14i |
| TR20 | 40.00±0.17i |
| TR44 | 44.76±0.34h |
| TR53 | 40.47±0.89i |

Supplementary Table 2: Percent inhibition of mycelial growth of *Bipolaris maydis* by *Trichoderma* isolates.

| Treatments | Root length | Shoot length | Fresh Root weight | Fresh Shoot weight | Dry shoot weight | Dry root weight | Total chlorophyll | Percent Disease Index |
| --- | --- | --- | --- | --- | --- | --- | --- | --- |
| T1 -CM212+E7+TR11 | 57.03^f^± | 113.33^a^± | 9.15^a^ | 25.57^a^ | 3.70a | 1.38a | 31.21a | 35.22c |
| T2 -CM212+E7(Pathogen treated control) | 42.43^e^ | 70.99^f^ | 7.13^d^ | 20.37^b^ | 2.75d | 0.52e | 20.34c | 44.74a |
| T3 -CM212) (Healthy control) | 49.10^d^ | 83.26^e^ | 8.04^b^ | 10.57^d^ | 2.35f | 0.67b | 21.40c | ------- |
| T4 -VL78+E7+TR11 | 52.10^b^ | 101.83^b^ | 7.65^c^ | 21.02^b^ | 3.46b | 0.57d | 31.14a | 31.88d |
| T5 -VL78+E7(Pathogen treated control) | 23.37^h^ | 71.50^f^ | 5.10^f^ | 14.36^c^ | 2.03g | 0.20f | 20.49c | 41.33b |
| T6 -VL78 (Healthy control) | 35.63^g^ | 85.00^d^ | 6.68^e^ | 20.33^b^ | 3.27c | 0.51e | 21.27c | --------- |
| T7 -Dhiari local +TR11+E7 | 57.06^a^ | 89.00^c^ | 7.48c | 14.74^c^ | 2.72d | 0.71b | 24.09b | 37.81c |
| T8 -Dhiari local+E7(Pathogen treated control) | 26.10^g^ | 58.23^h^ | 2.81^g^ | 6.12^e^ | 0.45h | 0.07g | 14.51e | 46.60a |
| T9 -Dhiari local healthy control) | 50.63^c^ | 66.03^g^ | 6.78^e^ | 14.81^c^ | 2.56e | 0.62c | 17.21d | --------- |

Supplementary Table 3: Effect of different treatments along with *Trichoderma* species on growth promotion and disease reduction on maize plants against *Bipolaris maydis*

Supplemantry Table 4: Changes in Superoxide dismutase (A), polyphenol oxidase (B), and Peroxidase (C) activities in Maize leaves on different treatments with *Trichoderma* isolates and challenged with *Bipolaris maydis* under glass-house conditions and observations were recorded after 25 days of sowing.

| SOD (Superoxide dismutase) | | | | |
| --- | --- | --- | --- | --- |
| Treatments | 0hr | 24hr | 48hr | 72hr |
| T1 | 0.32±0.01a | 0.82±0.01a | 1.24±0.02ab | 1.47±0.01a |
| T7 | 0.31±0.02a | 0.76±0.01b | 1.27±0.01a | 1.45±0.02a |
| T2 | 0.33±0.01a | 0.74±0.01c | 1.17±0.01c | 1.23±0.02c |
| T5 | 0.31±0.01b | 0.76±0.01b | 1.16±0.01c | 1.28±0.02b |
| T4 | 0.30±0.01b | 0.81±0.01a | 1.23±0.01b | 1.46±0.01a |
| T8 | 0.30±0.08b | 0.69±0.02d | 1.18±0.06c | 1.14±0.03d |
| T9 | 0.29±0.02bc | 0.29±0.02f | 0.29±0.01d | 0.29±0.01e |
| T6 | 0.29±0.01bc | 0.29±0.01f | 0.29±0.01d | 0.29±0.01e |
| T3 | 0.29±0.01bc | 0.29±0.01f | 0.29±0.01d | 0.29±0.01e |

| PO (Peroxidase) | | | | |
| --- | --- | --- | --- | --- |
| Treatments | 0hr | 24hr | 48hr | 72hr |
| T1 | 0.29±0.01a | 0.84±0.02a | 0.84±0.02a | 1.21±0.02a |
| T7 | 0.29±0.01a | 0.69±0.01d | 0.82±0.01a | 1.12±0.03b |
| T2 | 0.27±0.01b | 0.70±0.03d | 0.75±0.01b | 1.07±0.02c |
| T5 | 0.27±0.01b | 0.72±0.02c | 0.76±0.01b | 1.05±0.03d |
| T4 | 0.29±0.01a | 0.81±0.01b | 0.82±0.02a | 1.19±0.03a |
| T8 | 0.26±0.01c | 0.32±0.01e | 0.72±0.03c | 1.09±0.03b |
| T9 | 0.26±0.01c | 0.26±0.01f | 0.26±0.01d | 0.26±0.01e |
| T6 | 0.27±0.01b | 0.27±0.01f | 0.27±0.01d | 0.27±0.01e |
| T3 | 0.26±0.01c | 0.26±0.01f | 0.26±0.01d | 0.26±0.01e |

| PPO (Polyphenol oxidase) | | | | |
| --- | --- | --- | --- | --- |
| Treatments | 0hr | 24hr | 48hr | 72hr |
| T1 | 0.27±0.03a | 0.39±0.03a | 0.66±0.03a | 0.78±0.04a |
| T7 | 0.28±0.03ab | 0.35±0.05abc | 0.59±0.03b | 0.69±0.02bc |
| T2 | 0.24±0.02bc | 0.32±0.01bc | 0.52±0.03c | 0.65±0.03cd |
| T5 | 0.25±0.01ab | 0.31±0.03c | 0.51±0.03c | 0.64±0.03d |
| T4 | 0.25±0.01ab | 0.37±0.04ab | 0.61±0.02b | 0.72±0.03b |
| T8 | 0.25±0.01ab | 0.31±0.02c | 0.52±0.02c | 0.24±0.02e |
| T9 | 0.23±0.02c | 0.23±0.02d | 0.23±0.02d | 0.23±0.02e |
| T6 | 0.25±0.02ab | 0.25±0.02d | 0.25±0.02d | 0.25±0.02e |
| T3 | 0.25±0.02ab | 0.25±0.02d | 0.25±0.02d | 0.25±0.02e |
